# Effects of *IL-6* Polymorphisms on Individual Susceptibility to Allergic Diseases: A Systematic Review and Meta-Analysis

**DOI:** 10.3389/fgene.2022.822091

**Published:** 2022-03-16

**Authors:** Ying Yang, Jingxin Xiao, Lingling Tang, Bohan Wang, Xianhong Sun, Zhongchi Xu, Li Liu, Suofang Shi

**Affiliations:** ^1^ Affiliated Hospital of Nanjing University of Chinese Medicine, Nanjing, China; ^2^ Taizhou Hospital of Chinese Medicine, Taizhou, China; ^3^ Laboratory of Molecular Biology, Central Laboratory, Jiangsu Province Hospital of Chinese Medicine, Affiliated Hospital of Nanjing University of Chinese Medicine, Nanjing, China

**Keywords:** IL-6, allergic diseases, meta-analysis, polymorphism, allergic rhinitis, allergic asthma, atopic dermatitis

## Abstract

**Background:** Many studies have assessed the potential link between *interleukin-6* polymorphisms and susceptibility to allergic diseases. However, the results are still conflicting. Therefore, a comprehensive meta-analysis can not only resolve differences but also provide clues for future projects.

**Methods:** A systematic electronic search was conducted on the databases of Web of Science, PubMed, and Cochrane Library to retrieve all published studies. Revman and Stata software were used for statistical analysis.

**Results:** This meta-analysis included 11 studies. The results revealed that there was a statistically significant association between *IL-6* rs1800795 polymorphism and the risk of asthma and allergic rhinitis in the general population. Subgroup analyses demonstrated that rs1800795 affected allergic diseases risk in different populations.

**Conclusion:** Our findings suggested that *IL-6* rs1800795 was associated with allergic diseases susceptibility among Asians and Caucasians in opposite trends, and it might influence the risk of asthma and allergic rhinitis. None of the *IL-6* polymorphisms were shared risk variants of allergic diseases.

## Introduction

Allergic diseases, including allergic rhinitis, allergic asthma, and atopic dermatitis, often coexist in the same individual and are considered multifactorial inflammatory diseases (Blumenthal, 2012; Pinart et al., 2014; Ferreira et al., 2017). Allergic diseases have become major health problems worldwide, resulting in significant economic and social burdens (Asher et al., 2006; Nutten, 2015). The mechanism and pathological characteristics of allergic diseases are similar. Genes, environment factors, and the mutual effects among them can be the underlying mechanism of the progress and expression of these diseases (Blumenthal 2012; Galli and Tsai 2012; Ferreira et al., 2017). It shows that initial manifestations of atopic dermatitis often occur early in life and frequently ahead of other allergic diseases like asthma or allergic rhinitis (Nutten 2015), partially due to a shared genetic origin (Ferreira et al., 2017). In order to identify shared risk variants, the nature of individual genes recognized as susceptible factors for allergic diseases have been summarized, and the list of these genetic factors may expand significantly with the latest emergence of genome-wide association approaches (Vercelli 2008; Cheng et al., 2014; Ferreira et al., 2017).

Interleukin-6 (IL-6) is an effective pro-inflammatory cytokine, which can be released in response to immune attacks or tissue damages and stimulate a wide range of innate and adaptive immune responses (Tanaka et al., 2014). Overexpression of IL-6 can cause chronic inflammatory disorders and potentially fatal hyperinflammation (Spencer et al., 2019), as seen in advanced coronavirus disease 2019 (COVID-19) (Mehta et al., 2020). More and more evidence shows that IL-6 can modulate the differentiation and activation of T cells and induce the production of Th2 cytokines, which is an important signal for coordinating chronic inflammation and adaptive immunity (Rincon and Irvin 2012). Furthermore, IL-6 and transforming growth factor-β (TGF-β) together induce the production of Th17 cells while inhibiting the differentiation of Treg cells (Tanaka et al., 2014). Previous studies have found that IL-6 is related to the occurrence of allergic diseases such as asthma and atopic dermatitis (Gentile et al., 2001; Zhang et al., 2014), and it also has been shown that IL-6 can increase nasal secretion in patients with allergic rhinitis (Gentile et al., 2001).

Human IL-6 is composed of 212 amino acids, including a 28-amino-acid signal peptide, and its gene has been located on chromosome 7p21 (Kämäräinen et al., 2008); glycosylation accounts for the size of 21–26 kDa of natural IL-6 (Tanaka et al., 2014). Recent studies demonstrated the association between *IL-6* gene polymorphisms and allergic diseases risk. There are many verified single-nucleotide polymorphisms (SNPs) in the *IL6* gene in the dbSNP database, but only three SNPs, including −174 G/C (rs1800795), −572 G/C (rs1800796), and −597 G/A (rs1800797) restriction sites have been extensively studied (Kämäräinen et al., 2008; Cash et al., 2010). Many studies aimed to evaluate the relationship between the *IL-6* polymorphisms and allergic diseases risk in different populations (Reich et al., 2003; Settin et al., 2008; Trajkov et al., 2008; Xiaomin et al., 2008; Mahdaviani et al., 2009; Daneshmandi et al., 2012; Gharagozlou et al., 2013; Kosugi et al., 2013; Nasiri et al., 2014; Zhao et al., 2016; Babusikova et al., 2017). Nevertheless, the outcomes were still elusive. Hence, the purpose of the present research lies at generalizing the existing proof to clarify the exact association between *IL-6* gene polymorphisms and the risk of allergic diseases.

Previously, two meta-analyses examined the association between *IL-6* rs1800795 polymorphism and asthma risk (Li et al., 2015; Zhu et al., 2020). However, these two articles did not study the relationship between other allergic diseases with *IL6* gene polymorphisms and did not concern the two other polymorphisms of the *IL-6* (rs1800796 and rs1800797), although the associations between these SNPs and asthma have been reported. Thus, we performed this meta-analysis based on the accumulating evidence to estimate the link between *IL-6* gene polymorphisms and allergic diseases risk (including allergic rhinitis, allergic asthma, and atopic dermatitis). Besides these, we carried out isolated meta-analyses for each allergic disease to check the impact of *IL-6* SNPs on each disease and also to identify whether *IL-6* SNPs were shared risk variants of allergic diseases. Therefore, our meta-analysis was the first study on genetic associations of *IL-6* rs1800795, rs1800796, and rs1800797 polymorphisms with allergic diseases.

## Materials and Methods

Our research was carried out as per the Preferred Reporting Items for Systematic reviews and Meta-Analyses (PRISMA) guideline (Liberati et al., 2009).

### Literature Review and Inclusion Criteria

We used the keywords below to search for suitable studies from PubMed, Web of Science, and Cochrane Library: “IL-6,” “Interleukin-6,” “polymorphism,” “variant,” “variation,” “mutation,” “SNP,” “asthma,” “atopic dermatitis,” and “allergic rhinitis.” In February 2021, we conducted the initial literature search, and the recent renewal was completed in June 2021. Additionally, our team went through the bibliographies of the acquired studies to obtain more underlying related articles.

The selection standards of this study were as follows: 1) case–control study was designed to explore the association between *IL-6* SNPs and allergic diseases risk; 2) published research data can be adopted to speculate an odds ratio (OR) and 95% confidence interval (CI); and 3) full text was provided. If any of the standards below apply, the research will be excluded: 1) incomplete or inaccessible study data; 2) summaries or meta-analyses; and 3) research that was not based on human beings. In case that identical studies were accepted by several periodicals, we would merely include the latest complete studies for analysis.

### Data Extraction and Quality assessment

The entire necessary data were obtained from articles according to the normalized list of the information below: first author's name, publishing time, country, ethnicity, disease type, diagnosis basis, sample scale, genotype approach, and genotypical calculation for all polymorphic results. The *p*-value of Hardy–Weinberg equilibrium (HWE) was computed as well according to a formula. Our team adopted the Newcastle–Ottawa Scale (NOS) criteria to assess the quality of each study sequence (Zeng et al., 2015). According to the scores, studies were identified as inferior (0–3), ordinary (4–6), and superior (7–9) studies. Information obtainment and quality evaluation were carried out by two different researchers. Any disagreement between the two researchers was discussed till there was an agreement.

### Statistical Analysis

The entire statistic assays were analyzed *via* use of Revman 5.4 (The Nordic Cochrane Centre, Denmark) and Stata 15.1 (StataCorp LP, America). We used ORs and 95% CIs to assess the tightness of the connection between *IL-6* polymorphisms and allergic diseases risks. If *p* ≤ 0.05, it would be deemed as important on statistics. For all SNPs, the dominant comparison, recessive comparison, allele comparison, homozygote comparison, and heterozygote comparison were used to speculate the impacts. In detail, defined comparisons are as follows: for −174 G/C (rs1800795) and −572 G/C (rs1800796): dominant comparison (CC+GC *vs*. GG), recessive comparison (CC *vs*. CG+GG), allele comparison (C *vs*. G), heterozygote comparison (GC *vs*. GG), and homozygote comparison (CC *vs*. GG). For **−**597 G/A (rs1800797): dominant comparison (AA+GA *vs*. GG), recessive comparison (AA *vs*. AG+GG), allele comparison (A *vs*. G), heterozygote comparison (GA *vs*. GG), and homozygote comparison (AA *vs*. GG).

The heterogeneity between studies was assessed through use of the *I* ^2^ statistics. If *I* ^2^ > 50%, it meant that there was heterogeneity; the random-effects model was required to calculate the combination of OR. Otherwise, the fixed-effects model was employed (Higgins and Thompson 2002). Simultaneously, a χ^2^ test was adopted to test if the genotypical distribution of included studies accorded with the HWE, and *p* > 0.05 showed that it coincided. Moreover, the subgroup study was carried out as per ethnicity, age, and disease type. The steadiness of synthesized outcomes was assessed *via* sensitive analysis, and potential publication biases were assessed *via* funnel plots and Egger's test (Egger et al., 1997).

## Results

### Characteristics of Included Studies

**FIGURE 1:**
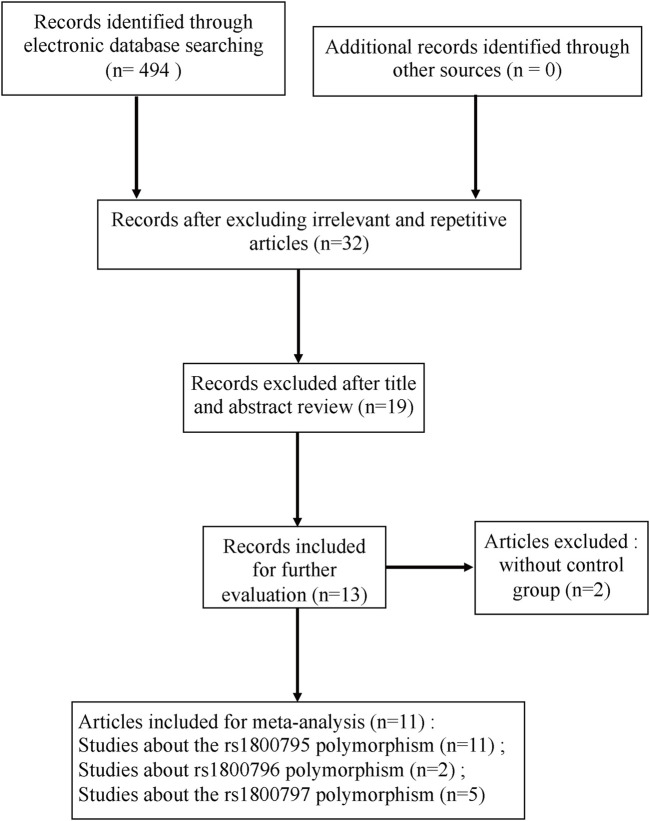
Flow diagram of the study selection process.

The search and screening process of this study is depicted in Figure 1 (Liberati et al., 2009). A total of 494 studies were selected, 462 studies were removed after excluding irrelevant and repetitive articles, then 32 articles were assessed for eligibility, and after reading headlines and abstracts, 19 articles were eliminated. Two others were excluded for lacking controls. Finally, a total of 11 eligible studies were included in this meta-analysis. The NOS score of the included studies was 7–8 points, which proved that the methodological quality of all included studies was generally good. In the included studies, PCR was used as a genotyping method. Tables 1 and 2 summarize the main characteristics of the included studies and the frequency of genotype distribution.

**TABLE 1:** Characteristics of the 11 case–control studies included in the meta-analysis.

First author	Year	Country		Ethnicity	Adults/children	IL-6 polymorphisms studied	Cases, n	Controls, n	Source of controls	Disease type	Disease definition	Genotyping method	NOS score
Liu	2008	China	Asians		Adults	rs1800795 rs1800797	108	88	PB	Asthma	Guidelines for Prevention and Treatment of Bronchial Asthma	PCR	7
Settin	2008	Egypt	Caucasian		Children	rs1800795	69	98	PB	Asthma	GINA criteria	PCR	7
Trajkov	2008	Macedonian	Caucasian		Adults	rs1800795	74	301	PB	Asthma	Criteria of National Institutes of Health	PCR	8
—	—	—	—		—	rs1800797	—	—	—	—	—	—	—
Mahdaviani	2009	Iran	Caucasian		Mixed	rs1800795	60	140	PB	Asthma	National Asthma Education and Prevention program	PCR	7
—	—	—	—		—	rs1800797	—	—	—	—	—	—	—
Kosugi	2012	Brazil	Mixed		Adults	rs1800795	90	144	PB	Asthma	Clinical and spirometry criteria	PCR	8
Babusikova	2016	Eslovaquia	Caucasian		Children	rs1800795 rs1800796	264	250	PB	Asthma	GINA criteria	PCR	8
Daneshmandi	2012	Iran	Caucasian		Adults	rs1800795	81	124	PB	Asthma	GINA criteria	PCR	7
Zhao	2016	China	Asians		Mixed	rs1800795	265	265	PB	Allergic rhinitis	Guidelines of the American Thoracic Society	PCR	8
—	—	—	—		—	rs1800796	—	—	—	—	—	—	—
Nasiri	2014	Iran	Caucasian		Mixed	rs1800795 rs1800797	88	139	PB	Allergic rhinitis	EAACI criteria	PCR	7
Gharagozlou	2013	Iran	Caucasian		Children	rs1800795	89	139	PB	Atopic dermatitis	Standard criteria	PCR	7
—	—	—	—		—	rs1800797	—	—	—	—	—	—	—
Reich	2003	Germany	Caucasian		Mixed	rs1800795	94	214	PB	Atopic dermatitis	Modified Hannifin and Rajka diagnostic criteria for AD	PCR	8

Abbreviations: PB, population-based controls; PCR, polymerase chain reaction; NOS: Newcastle–Ottawa Scale; GINA, Global Initiative for Asthma; EAACI, European Academy of Allergy and Clinical Immunology; AD, Alzheimer's disease.

**TABLE 2:** Distribution of the *IL-6* genotype among cases and controls included in this meta-analysis.

	First author	Cases			Controls			HWE *p*-value
		CC	CG	GG	CC	CG	GG	
−174 G/C (rs1800795)	Reich	19	48	27	44	104	66	0.80
	Gharagozlou	4	22	63	4	93	42	<0.001
	Nasiri	12	28	58	4	93	42	<0.001
	Zhao	55	122	88	40	118	107	0.43
	Mahdaviani	1	51	5	4	93	42	<0.001
	Liu	0	36	72	0	17	71	0.32
	Trajkov	3	25	46	25	132	144	0.49
	Settin	6	52	11	6	87	5	<0.001
	Daneshmandi	6	15	60	9	32	83	0.03
	Kosugi	1	20	69	7	67	70	0.07
	Babusikova	43	121	100	73	127	50	0.70

	First author	Cases			Controls			HWE *p*-value
		CC	CG	GG	CC	CG	GG	
−572 G/C (rs1800796)	Zhao	52	114	99	40	118	107	0.429
	Babusikova	11	48	205	6	56	188	0.459

	First author	Cases			Controls			HWE *p*-value
		CC	CG	GG	CC	CG	GG	
−597 G/A (rs1800797)	Gharagozlou	4	22	63	4	42	93	0.775
	Nasiri	10	28	60	4	42	93	0.775
	Mahdaviani	0	18	40	4	42	93	0.775
	Liu	0	0	108	0	0	88	NA
	Trajkov	3	25	46	25	123	153	0.968

Abbreviations: HWE, Hardy–Weinberg equilibrium.

### Meta-Analysis of the Association Between −174 G/C rs1800795 Polymorphism and Overall Allergic Diseases Risk

**FIGURE 2:**
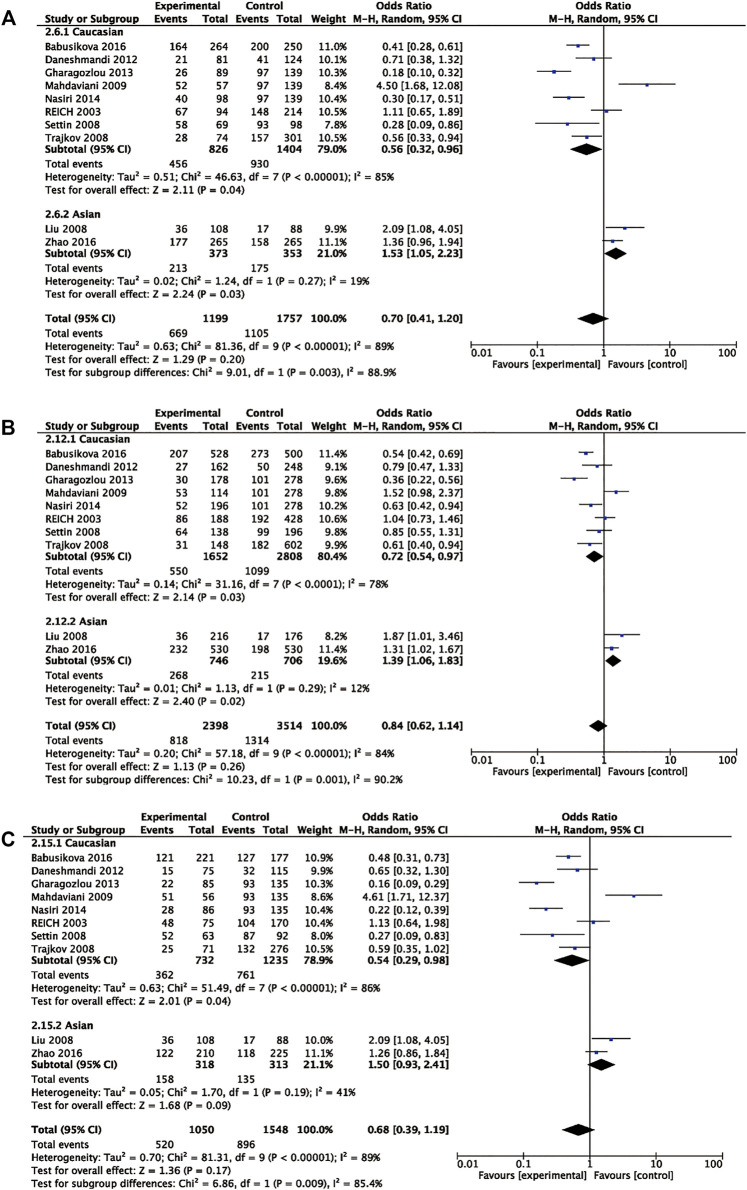
Pooled OR and 95% CI of individual studies and pooled data for the association between *IL6* rs1800795 and allergic diseases risk in different ethnicity subgroups for **(A)** dominant comparison, **(B)** allele comparison, and **(C)** heterozygote comparison.

Eleven articles included 1,282 patients with allergic disease, and 1,902 controls were analyzed to find the relationship between rs1800795 polymorphism and overall allergic diseases risk. Our results did not show significant association of rs1800795 polymorphism and overall allergic diseases risk in the general population (Table 3). However, subgroup analyses based on ethnicities demonstrated that rs1800795 polymorphism was associated with the decreased risk of overall allergic diseases in Caucasian populations under dominant comparison, allele comparison, and heterozygote comparison (dominant comparison: OR = 0.56, 95% CI =0.32, 0.96, *p* = 0.04; allele comparison: OR = 0.72, 95% CI = 0.54, 0.97, *p* = 0.03; heterozygote comparison: OR = 0.54, 95% CI =0.29, 0.98, *p* = 0.04), while in Asian populations, rs1800795 polymorphism was associated with an increased risk of overall allergic diseases in dominant comparison, allele comparison, and homozygote comparison (dominant comparison: OR = 1.53, 95% CI =1.05, 2.23, *p* = 0.03; allele comparison: OR = 1.39, 95% CI =1.06, 1.83, *p* = 0.02; homozygote comparison: OR = 1.67, 95% CI =1.02, 2.74, *p* = 0.04) (Table 3 and Figure 2).

**TABLE 3:** Results of pooled ORs in the meta-analysis of the association between *IL-6* gene polymorphisms and overall allergic disease.

	Population	Sample size, cases/controls	Dominant comparison			Recessive comparison			Allele comparison			Heterozygote comparison			Homozygote comparison		
			*p*-value	OR (95%CI)	*I* ^2^ statistic	*p*-value	OR (95%CI)	*I* ^2^ statistic	*p*-value	OR (95%CI)	*I* ^2^ statistic	*p*-value	OR (95%CI)	*I* ^2^ statistic	*p*-value	OR (95%CI)	*I* ^2^ statistic
**−174 G/C (rs1800795)**	Overall	1282/1902	0.09	0.63 (0.38, 1.07)	89%	1	1.00 (0.61, 1.63)	65%	0.11	0.78 (0.58, 1.06)	85%	0.08	0.63 (0.37, 1.06)	89%	0.34	0.75 (0.41, 1.36)	72%
	Caucasian	819/1405	**0.04**	**0.56 (0.32, 0.96)**	**85%**	1	1.00 (0.57, 1.74)	61%	**0.03**	**0.72 (0.54, 0.97)**	**78%**	**0.04**	**0.54 (0.29, 0.98)**	86%	0.24	0.70 (0.39, 1.27)	58%
	Asians	373/353	**0.03**	**1.53 (1.05, 2.23)**	**19%**	0.09	1.47 (0.94, 2.31)	NA	**0.02**	**1.39 (1.06, 1.83)**	**12%**	0.09	1.50 (0.93, 2.41)	41%	**0.04**	**1.67 (1.02, 2.74)**	NA
	Children	422/487	*p* **< 0.0001**	**0.28 (0.16, 0.51)**	63%	0.75	0.86 (0.35, 2.14)	62%	**0.004**	**0.55 (0.36, 0.83)**	**72%**	**0.002**	**0.28 (0.13, 0.62)**	**77%**	***P* < 0.00001**	**0.33 (0.21, 0.52)**	**0**
	Adults	353/657	0.32	0.66 (0.29, 1.50)	85%	0.22	0.62 (0.28, 1.34)	4%	0.33	0.74 (0.40, 1.36)	82%	0.35	0.70 (0.32, 1.50)	84%	**0.04**	**0.47 (0.23, 0.97)**	**28%**
**−572 G/C (rs1800796)**	Overall	529/515	0.91	1.02 (0.78, 1.32)	0	0.09	1.43 (0.95, 2.17)	0	0.34	1.10 (0.90, 1.35)	0	0.59	0.92 (0.70, 1.23)	0	0.1	1.46 (0.93, 2.27)	0
**−597 G/A** **(rs1800797)**	Overall	419/807	0.36	0.88 (0.66, 1.16)	14%	0.8	1.16 (0.36, 3.74)	58%	0.72	0.94 (0.65, 1.35)	55%	0.25	0.84 (0.63, 1.13)	0	0.91	1.08 (0.31, 3.71)	62%
	Caucasian	311/719	0.36	0.88 (0.66, 1.16)	14%	0.8	1.16 (0.36, 3.74)	58%	0.72	0.94 (0.65, 1.35)	55%	0.25	0.84 (0.63, 1.13)	0	0.91	1.08 (0.31, 3.71)	62%
	Adults	182/389	0.08	0.63 (0.37, 1.06)	NA	0.22	0.47 (0.14, 1.59)	NA	0.06	0.66 (0.43, 1.01)	NA	0.16	0.68 (0.39, 1.16)	NA	0.15	0.40 (0.12, 1.38)	NA

Abbreviations: OR, odds ratio; CI, confidence interval; NA, not available. The values in bold denote that there are statistically significant differences between cases and controls.

**FIGURE 3:**
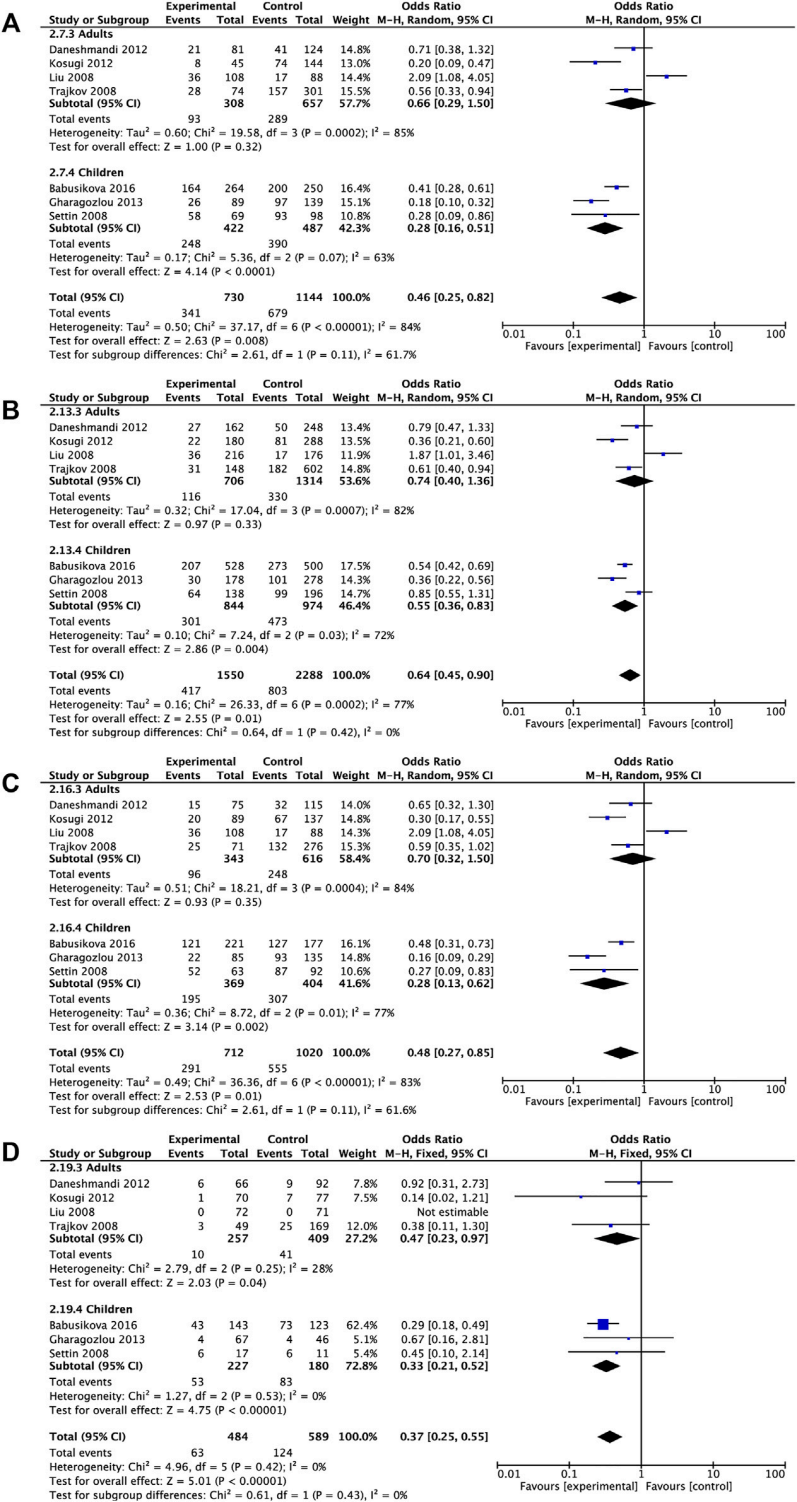
Pooled OR and 95% CI of individual studies and pooled data for the association between *IL6* rs1800795 polymorphism and overall allergic diseases risk in different age subgroups for **(A)** dominant comparison, **(B)** allele comparison, **(C)** heterozygote comparison, and **(D)** homozygote comparison.

Subgroup analyses on the basis of age demonstrated that *IL6* rs1800795 variant was associated with a decreased risk of childhood allergic diseases in dominant comparison, allele comparison, heterozygote comparison, and homozygote comparison (dominant comparison: OR = 0.28, 95% CI = 0.16, 0.51, *p* < 0.0001, allele comparison: OR = 0.55, 95% CI = 0.36, 0.83, *p* = 0.004, heterozygote comparison: OR = 0.28, 95% CI = 0.13, 0.62, *p* = 0.002, homozygote comparison: OR = 0.33, 95% CI = 0.21, 0.52, *p* < 0.00001). The decreased risk of allergic diseases was related to rs1800795 polymorphism across homozygote comparison (homozygote comparison: OR = 0.47, 95% CI = 0.23, 0.97, *p* = 0.04) (Table 3 and Figure 3).

### Meta-Analysis of the Association Between −572 G/C rs1800796 Polymorphism and Overall Allergic Diseases Risk

Two articles including 529 patients and 515 controls were analyzed to investigate the association between rs1800796 polymorphism and overall allergic diseases risk. Of the two articles, one was about asthma, reporting 264 patients and 250 controls, and the other was about allergic rhinitis, including 265 patients and 265 controls. Since one study was on Caucasian children and the other was on mixed-age Asians, subgroup analysis based on age and ethnicity was not feasible. The pooled analyses indicated that under any comparison, there was no significant association between the rs1800796 polymorphism and the risk of allergic diseases (Table 3). This negative result may be due to insufficient research, so more research was needed to corroborate our conclusion.

### Meta-Analysis of the Association Between nt565 G/A rs1800797 Polymorphism and Overall Allergic Diseases Risk

Five articles including 419 patients and 807 controls were analyzed to find the association between rs1800797 polymorphism and allergic diseases risk. Among the five articles, three were about asthma, one about allergic rhinitis, and one about atopic dermatitis. In any of our comparison analyses, no significant association was found between the rs1800797 polymorphism and the risk of overall allergic diseases (Table 3). Further stratification analysis by ethnicity and age revealed that no significantly decreased or increased risks of overall allergic diseases were found in any genetic model (Table 3). This result may still be caused by the lack of research, which needs more research to confirm.

### Meta-Analysis of the Association Between *IL-6* Polymorphisms and the Risk of Each Allergic Disease

Considering the different pathogenic and epigenetic potential factors of the three allergic diseases, the relationship between *IL-6* gene polymorphisms and the risk of each disease may be different. Therefore, it is necessary to study each form of allergy separately and classify them separately to test the effectiveness of the study in each category.

**FIGURE 4:**
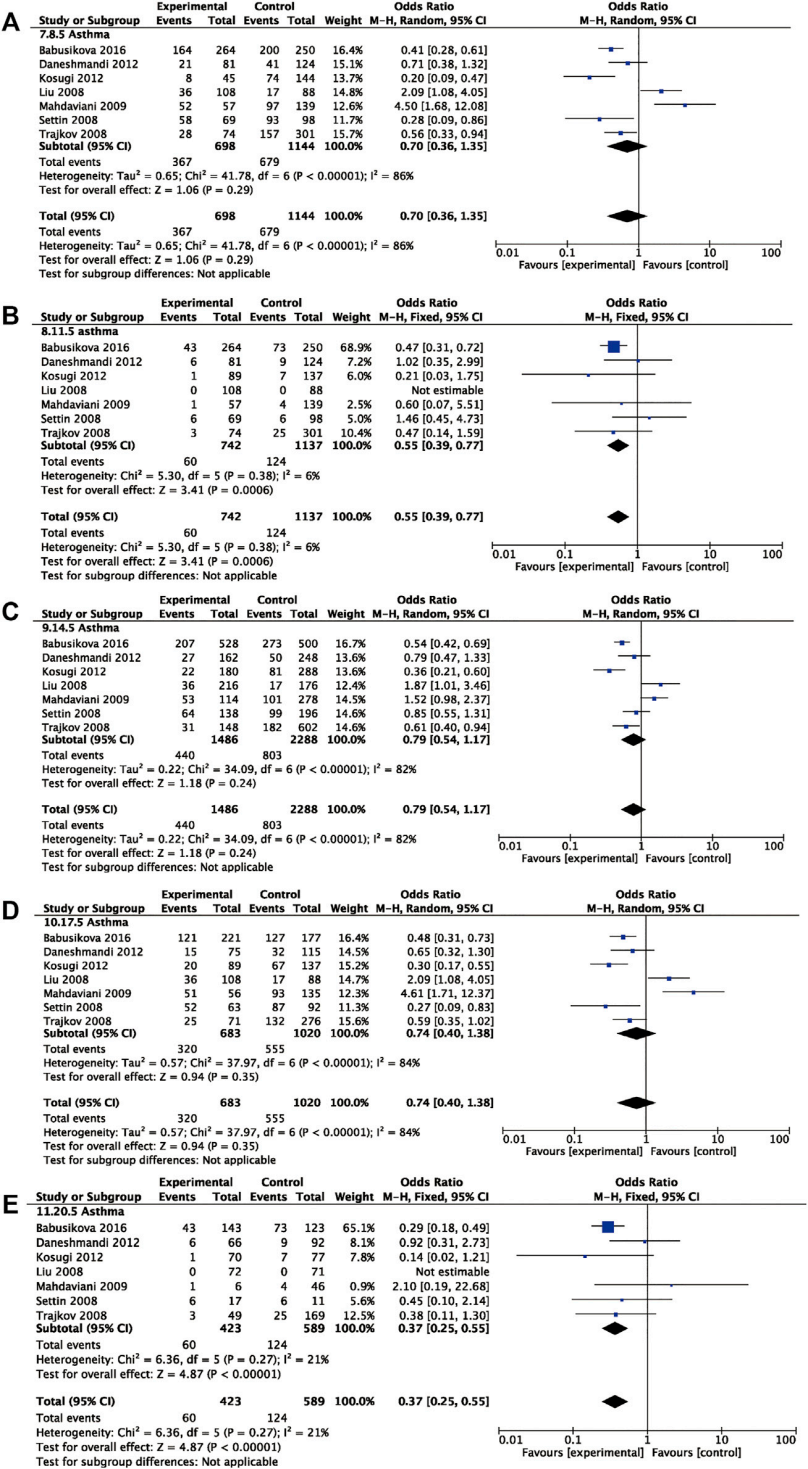
Pooled OR and 95% CI of individual studies and pooled data for the association between *IL-6* rs1800795 polymorphism and asthma risk for **(A)** dominant comparison, **(B)** recessive comparison, **(C)** allele comparison, **(D)** heterozygote comparison, and **(E)** homozygote comparison.

Seven studies involving 746 asthmatic patients and 1,145 controls were analyzed to determine the association of rs1800795 and asthma risk. The results revealed that rs1800795 polymorphism was significantly associated with the risk of asthma under the recessive comparison and homozygote comparison (recessive comparison: OR = 0.55, 95% CI = 0.39, 0.77, *p* = 0.0006, homozygote comparison: OR = 0.37, 95% CI = 0.25, 0.55, *p*< 0.00001) (Table 4 and Figure 4).

**TABLE 4:** Results of pooled ORs in the meta-analysis of the association between *IL-6* gene polymorphisms and each allergic disease.

Polymorphism and disease	Population	Sample size, cases/controls	Dominant comparison			Recessive comparison			Allele comparison			Heterozygote comparison			Homozygote comparison		
			*p*-value	OR (95%CI)	*I* ^2^ statistic	*p*-value	OR (95%CI)	*I* ^2^ statistic	*p*-value	OR (95%CI)	*I* ^2^ statistic	*p*-value	OR (95%CI)	*I* ^2^ statistic	*p*-value	OR (95%CI)	*I* ^2^ statistic
−174 G/C (rs1800795) and asthma	Overall	746/1145	0.29	0.70 (0.36, 1.35)	86%	**0.0006**	**0.55 (0.39, 0.77)**	**6%**	0.24	0.79 (0.54, 1.17)	82%	0.35	0.74 (0.40, 1.38)	84%	***p* < 0.00001**	**0.37 (0.25, 0.55)**	**21%**
	Caucasian	548/913	0.30	0.70 (0.35, 1.37)	82%	**0.002**	**0.57 (0.40, 0.81)**	**10%**	0.22	0.79 (0.54, 1.15)	77%	0.34	0.72 (0.37, 1.42)	80%	***p* < 0.00001**	**0.39 (0.26, 0.58)**	**28%**
	Children	333/348	***p* < 0.00001**	**0.39 (0.27, 0.57)**	0	0.55	0.72 (0.25, 2.11)	68%	0.06	0.65 (0.42, 1.01)	69%	***p* < 0.00001**	**0.44 (0.30, 0.66)**	84%	***p* < 0.00001**	**0.31 (0.19, 0.50)**	**0**
	Adults	353/657	0.11	0.66 (0.29, 1.50)	85%	0.22	0.62 (0.28, 1.34)	4%	0.33	0.74 (0.40, 1.36)	82%	0.35	0.70 (0.32, 1.50)	0	**0.04**	**0.47 (0.23, 0.97)**	**28%**
−174 G/C (rs1800795) and allergic rhinitis	Overall	353/404	0.57	0.65 (0.15, 2.86)	95%	0.14	2.32 (0.76, 7.04)	70%	0.83	0.93 (0.46, 1.88)	89%	0.47	0.53 (0.10, 2.96)	96%	**0.02**	**1.74 (1.10, 2.75)**	**0**
−174 G/C (rs1800795) and atopic dermatitis	Overall	183/353	0.38	0.45 (0.07, 2.67)	95%	0.85	1.05 (0.61, 1.84)	0	0.36	0.61 (0.21, 1.75)	92%	0.38	0.42 (0.06, 2.91)	95%	0.92	0.97 (0.52, 1.82)	0
−597 G/A (rs1800797) and asthma	Overall	242/529	0.12	0.72 (0.48, 1.09)	0	0.14	0.43 (0.14, 1.32)	0	0.06	0.72 (0.50, 1.01)	0	0.26	0.79 (0.52, 1.20)	0	0.09	0.37 (0.12, 1.16)	0
	Caucasian	134/441	0.12	0.72 (0.48, 1.09)	0	0.14	0.43 (0.14, 1.32)	0	0.06	0.72 (0.50, 1.01)	0	0.26	0.79 (0.52, 1.20)	0	0.09	0.37 (0.12, 1.16)	0

Abbreviations: OR, odds ratio; CI, confidence interval. The values in bold denote that there are statistically significant differences between cases and controls.

Further subgroup analyses were used for ethnicity and age. The rs1800795 polymorphism decreased the risk of asthma in Caucasians across the recessive comparison and homozygote comparison (recessive comparison: OR = 0.57, 95% CI = 0.40, 0.81, *p* = 0.002, homozygote comparison: OR = 0.39, 95% CI = 0.26, 0.58, *p* < 0.00001) and was associated with a decreased risk of asthma in children in the dominant comparison, heterozygote comparison, and homozygote comparison (dominant comparison: OR = 0.39, 95% CI = 0.27, 0.57, *p* < 0.0001; heterozygote comparison: OR = 0.44, 95% CI = 0.30, 0.66, *p* < 0.0001, homozygote comparison: OR = 0.31, 95% CI = 0.19, 0.50, *p* < 0.00001). The same trend was found in the adults under the homozygote comparison (homozygote comparison: OR = 0.47, 95% CI = 0.23, 0.97, *p* = 0.04) (Table 4).

Two case–control studies including 353 patients with allergic rhinitis and 404 controls were utilized to analyze the relationship between *IL-6* rs1800795 and allergic rhinitis. The result showed that *IL-6* rs1800795 polymorphism increased the risk for allergic rhinitis in homozygote comparison (homozygote comparison = 1.74, 95% CI = 1.10, 2.75, *p* = 0.02).

Two case–control studies, including 183 patients and 353 controls, were utilized to analyze the association between rs1800795 and atopic dermatitis. The results showed that there was no significant association between *IL-6* rs1800795 polymorphism and atopic dermatitis risk (Table 4).

Three case–control studies, including 242 asthmatic patients and 529 controls, were utilized to analyze the association between rs1800797 and asthma. The results showed no association between the polymorphism and risk of asthma in any of the models (Table 4).

### Publication Bias

Egger's test and Funnel plots were performed to measure the publication bias. The funnel plots' shapes of rs1800795 did not show any evidence of obvious asymmetry. Meanwhile, the results of Egger's tests also showed no evidence of publication biases. The associative outcomes of rs1800796 and 1800797 were less than 10 studies, so they were precluded from publication bias testing (Supplementary Figures S8 and S9) .

### Sensitivity Analysis

We performed sensitivity analysis by deleting one eligible study at a time (Supplementary Figures S4–S7). The significance of the pooled ORs was not affected by any single study in each comparison for rs1800796, rs1800797, while for rs1800795, the combined ORs of some comparisons were changed significantly after removing Mahdaviani's (Mahdaviani et al., 2009), Babusikova's (Babusikova et al., 2017), and Liu's (Xiaomin et al., 2008) studies in the general, adult, and Caucasian populations. We also excluded the studies with a deviation of HWE in the controls, and some results changed, which indicated that the results of these comparisons were controversial and needed more studies to verify in the future (Supplementary Tables S2 and S3).

## Discussion

Human genome research emphasizes the role of common genetic variations in the etiology and pathogenesis of allergic diseases (Asher et al., 2006; Nutten 2015). The *IL-6* gene has three promoter variants, rs1800795, rs1800796, and rs1800797, which have been proved to affect the transcription and secretion of IL-6 and are suspected to be risk factors for allergic diseases (Fishman et al., 1998; Kämäräinen et al., 2008). Considering the inconsistent results of published studies, we included all eligible publications for the meta-analysis to assess the link between *IL-6* polymorphisms and the risk of allergic diseases.

Excluding the results with poor stability, our findings indicated no evidence of the association of *IL-6* rs1800795, rs1800796, or rs1800797 polymorphism with overall allergic diseases risk in general study populations. However, in the ethnicity-based subgroup analysis, the presence of rs1800795 SNP in Caucasians reduced the risk of allergic diseases but elevated the risk of allergic diseases in Asians. The trends of association in Asians and Caucasians were contrary to each other, indicating that there might be large differences in the genotype distribution of rs1800795 polymorphism among different ethnicities. These may be due to geographical differences, distinct dietary habits, ethnic diversity, the influence of ethnicity on *IL-6* gene expression, and serum IL-6 levels. Stratified by age, *IL-6* rs1800795 was negatively related to susceptibilities of overall allergic diseases in both adults and children.

When we studied the association between *IL-6* gene polymorphisms and each allergic disease separately, we found that the rs1800795 polymorphism was associated with a lower risk of asthma but was associated with increased susceptibility to allergic rhinitis. The age-based and ethnicity-based subgroup analyses in asthma indicated that *IL-6* rs1800795 was a protective factor against asthma in Caucasians—children and adults.

We included allergic rhinitis, atopic dermatitis, and asthma in this study, hoping to explore whether *IL-6* polymorphisms were the common risk variants of allergic diseases. According to our results, the rs1800795 polymorphism had the opposite effect on the susceptibility to asthma and allergic rhinitis and had no effect on the risk of atopic dermatitis. *IL-6* rs1800796 and rs1800797 polymorphisms were not related to the risk of allergic diseases. All of these results indicated that *IL-6* polymorphisms might not be the shared risk variants of allergic diseases. This conclusion may not be accurate enough due to the lack of studies, since there were only two studies on allergic rhinitis and two on atopic dermatitis.

It is known that the pathogenesis of allergic diseases involves the complex interaction of genetic and environmental factors, and ethnicities seem to have a significant impact on the prevalence of allergic diseases (Kim et al., 2020). It is reported that compared to the Asians, asthma risk was significantly greater for Black/African American, American Indian and Alaska Native (AIAN), White, and multiracial respondents (Hall et al., 2020). A study from the Netherlands found that Caucasians were less likely to have allergic rhinitis than most other ethnicities (Hoffmans et al., 2018). The above prompted us that the morbidity of asthma and allergic rhinitis showed opposite trends in Caucasians and Asians, which was consistent with our result that the association between *IL-6* rs1800795 polymorphism and the risk of allergic disease showed opposite trends in different ethnicities. We speculate that the *IL-6* rs1800795 polymorphism may be the same as factors such as residence, lifestyle, and eating habits (Hoffmans et al., 2018; Hall et al., 2020), which is the reason for the different susceptibilities of different ethnic groups to asthma and allergic rhinitis. However, more studies are still needed to prove this.

*IL-6* rs1800795 polymorphism was reported to be close to the sites of many transcription factors, including NF-κB and NF-IL6. Guanine-containing oligonucleotides at position −174 have more DNA protein interactions than cytosine-containing oligonucleotides (Ray et al., 1990; Terry et al., 2000). Therefore, they have a higher affinity for transcription factors, resulting in increased *IL-6* gene expression (Fishman et al., 1998). IL-6 has emerged as an important regulator of effector CD4 T cell fate, promoting IL-4 production during Th2 differentiation, inhibiting Th1 differentiation, and, together with TGF-β, promoting Th17 cell differentiation (Zhu et al., 2010; Rincon and Irvin 2012). Based on these, we hypothesized that rs1800795 polymorphism could affect the differentiation of Th1 and Th17 cells by affecting the expression of *IL-6*, thereby affecting the risk of allergic diseases.

It is reported that rs1800796 does not contain any effective homology with any identified transcription factor binding site; this may be why rs1800796 was not associated with the risk of allergic diseases. The *IL-6* rs1800797 polymorphism is another mutation in the *IL-6* gene promoter region. Studies have shown that it can regulate the effect of rs1800795 on transcriptional activity (Terry et al., 2000; Rivera-Chavez et al., 2003), but our study did not reflect the influence of rs1800797 on the risk of allergic diseases.

As for evaluation of heterogeneities, for *IL-6* rs1800796 and rs1800797 polymorphisms, we observed that heterogeneities were slight. However, moderate to severe heterogeneities were observed among eligible studies in certain comparisons for rs1800795. In the further subgroup analysis, we found that the heterogeneity of rs1800795 was decreased in Asians, Caucasians, children, and adults, which indicated that distributions of rs1800795 polymorphism varies greatly from population to population. Moreover, differences in ethnicity and age could affect genetic association between *IL6* rs1800795 polymorphism and allergic diseases. Therefore, the genetic associations between rs1800795 polymorphism and allergic diseases may be ethnic-specific and age-specific.

There are several points to consider in our findings. Firstly, in the sensitivity analysis, we found that the removal of Liu's (Li et al., 2015) study would have a significant effect on the results in the overall population. Considering that the study of Liu was in line with HWE, it might be that the difference in ethnicity caused this poor stability. The significantly reduced heterogeneity and sensitivity in the ethnicity-based subgroup analysis confirmed this. So the genetic associations between polymorphisms in *IL-6* and allergic diseases risk may be ethnically specific, and maybe we should not try to generalize the combined results of our positive findings to a wider population. Secondly, the pathogenesis of allergic diseases is very complex. Therefore, our team highly suggests that other studies should carry out monomer-type studies and explore underlying gene–gene mutual effects to further reveal the impacts of genes on allergic diseases susceptibility (Steen 2012; Nishi et al., 2016). Thirdly, we aimed to investigate more allergic diseases such as eczema or urticaria or allergic conjunctivitis at the beginning. However, we did not find studies on the relationship between these diseases and *IL-6* gene polymorphisms. Moreover, there is also a lack of research on allergic rhinitis and atopic dermatitis. Maybe scholars should pay more attention to relative allergic diseases in the future.

Previous relevant meta-analyses were reported by Zhu et al. (2020) and Li et al. (2015), which were partly consistent with our results in the association between *IL-6* rs1800795 and asthma risk. In addition, our study has been expanded in the following aspects. Through the inclusion of studies on three kinds of allergic diseases and three *IL-6* polymorphisms, we explored whether the *IL-6* SNPs were shared risk variants of these allergic diseases. Moreover, we conducted subgroup analyses on ethnicity, age, and disease type. Hence, our findings seem to be more convincing and abundant.

Our research also had some unavoidable defects. First of all, only 11 studies were analyzed, and the specimen scale was very limited, resulting in insufficient outcome dependability. Second, environmental factors might impact the association between *IL-6* polymorphisms and allergic diseases as well. Unfortunately, the majority of selected studies merely highlight the association, so it is hard to analyze the gene–environment mutual effects. Third, *IL-6* polymorphisms may be associated with the severity and susceptibility of these diseases, but we did not perform a meta-analysis on this, owing to the lack of relevant data in the included studies. Fourth, the sample size of the current study is still insufficient, especially in the subgroup analysis. Therefore, large and well-designed studies are needed to validate our findings. Fifth, we excluded the gray literature from the analysis because its quality is difficult to evaluate. Considering the above mentioned limitations, our findings should be interpreted with caution.

## Conclusion

This meta-analysis demonstrated that rs1800795 polymorphism of *IL-6* gene might be a risk factor for allergic rhinitis. In addition, *IL-6* rs1800795 polymorphism was associated with allergic diseases susceptibility among Asians. However, the opposite trend appeared in Caucasians. *IL-6* rs1800795 polymorphism was a protective factor against asthma, while it was associated with increased susceptibility to allergic rhinitis. Neither rs1800795, rs1800796, nor rs1800797 was a shared risk variant of overall allergic diseases. However, further well-designed studies with a bigger specimen scale are needed to verify the discoveries in this work. Additionally, more studies are required as well to reveal the possible molecular mechanisms of the discoveries in this article.

## Data Availability

The original contributions presented in the study are included in the article/Supplementary Material, further inquiries can be directed to the corresponding authors.

## Abbreviations

CI, confidence interval; IL, interleukin; NOS, Newcastle–Ottawa Scale; OR, odds ratio; PRISMA, Preferred Reporting Items for Systematic reviews and Meta-Analyses; SNP, single-nucleotide polymorphism; TGF-β, transforming growth factor-β.
