# Supplementary material for: Effects of IL-6 Polymorphisms on Individual Susceptibility to Allergic Diseases: A Systematic Review and Meta-Analysis
Source: Front Genet. 2022 Mar 16;13:822091. doi: 10.3389/fgene.2022.822091 (PMC8966614; doi:10.3389/fgene.2022.822091)
Supplement: Supplementary file 1 [file DataSheet1.pdf]

Supplementary Materials for "Effects of *IL-6* polymorphisms on individual susceptibility to Allergic Diseases: A Systematic Review and Meta-analysis"

Supplementary Table 1. Search strategies.

Supplementary Table 2. Main results of pooled ORs in the meta-analysis of IL-6 gene polymorphisms（After removing the studies of deviation from HWE）.

Supplementary Table 3. Main results of pooled ORs in the meta-analysis of IL-6 gene polymorphisms（After removing the studies of deviation from HWE）.

Supplementary Table 4. *P* value of Egger's tests for *IL-6* rs1800795 polymorphism and allergic diseases risk under the A: Dominant comparison; B: Recessive comparison; C: Allele comparison; D: Heterozygote comparison; E: Homozygote comparison.

Supplementary Figure 1. Pooled OR and 95% CI of individual studies and pooled data for the association between *IL-6* rs1800795 polymorphism and allergic diseases risk under the A: Dominant comparison; B: Recessive comparison; C: Allele comparison; D: Heterozygote comparison; E: Homozygote comparison.

Supplementary Figure 2. Pooled OR and 95% CI of individual studies and pooled data for the association between *IL-6* rs1800796 polymorphism and allergic diseases risk under the A: Dominant comparison; B: Recessive comparison; C: Allele comparison; D: Heterozygote comparison; E: Homozygote comparison.

Supplementary Figure 3. Pooled OR and 95% CI of individual studies and pooled data for the association between *IL-6* rs1800797 polymorphism and allergic diseases risk under the A: Dominant comparison; B: Recessive comparison; C: Allele comparison; D: Heterozygote comparison; E: Homozygote comparison.

Supplementary Figure 4. Sensitivity analyses of *IL-6* rs1800795 and allergic diseases risk under the A: Dominant comparison; B: Recessive comparison; C: Allele comparison; D: Heterozygote comparison; E: Homozygote comparison.

Supplementary Figure 5. Sensitivity analyses of *IL-6* rs1800797 and allergic diseases risk under the A: Dominant comparison; B: Recessive comparison; C: Allele comparison; D: Heterozygote comparison; E: Homozygote comparison.

Supplementary Figure 6. Sensitivity analyses of *IL-6* rs1800795 and asthma risk under the A: Dominant comparison; B: Recessive comparison; C: Allele comparison; D: Heterozygote comparison; E: Homozygote comparison.

Supplementary Figure 7. Sensitivity analyses of *IL-6* rs1800797 and asthma under the A: Dominant comparison; B: Recessive comparison; C: Allele comparison; D: Heterozygote comparison; E: Homozygote comparison.

Supplementary Figure 8. Funnel plots of *IL6* rs1800795 and allergic diseases risk under the A: Dominant Model; B: Recessive comparison; C: Allele comparison; D: Heterozygote comparison; E: Homozygote comparison.

Supplementary Figure 9. Egger's tests for *IL-6* rs1800795 and allergic diseases risk under the A: Dominant comparison; B: Recessive comparison; C: Allele comparison; D: Heterozygote comparison; E: Homozygote comparison.

Supplementary Figure 10. The progress of Egger's tests for *IL-6* rs1800795 and allergic diseases risk under the A: Dominant comparison; B: Recessive comparison; C: Allele comparison; D: Heterozygote comparison; E: Homozygote comparison.

Supplementary Table 1. Search strategies.

Databases	Search items	Result
PubMed	("asthma"[MeSH Terms] OR "asthma"[All Fields] OR "asthmas"[All Fields] OR "asthma s"[All Fields] OR ("asthma"[MeSH Terms] OR "asthma"[All Fields] OR "asthmatic"[All Fields] OR "asthmatics"[All Fields]) OR ("dermatitis, atopic"[MeSH Terms] OR ("dermatitis"[All Fields] AND "atopic"[All Fields]) OR "atopic dermatitis"[All Fields] OR ("atopic"[All Fields] AND "dermatitis"[All Fields])) OR ("rhinitis, allergic"[MeSH Terms] OR ("rhinitis"[All Fields] AND "allergic"[All Fields]) OR "allergic rhinitis"[All Fields] OR ("allergic"[All Fields] AND "rhinitis"[All Fields]))) AND (("interleukin 6"[MeSH Terms] OR "interleukin 6"[All Fields] OR "il 6"[All Fields] OR ("interleukin 6"[MeSH Terms] OR "interleukin 6"[All Fields] OR "interleukin 6"[All Fields])) AND ("polymorphic"[All Fields] OR "polymorphics"[All Fields] OR "polymorphism s"[All Fields] OR "polymorphism, genetic"[MeSH Terms] OR ("polymorphism"[All Fields] AND "genetic"[All Fields]) OR "genetic polymorphism"[All Fields] OR "polymorphism"[All Fields] OR "polymorphisms"[All Fields] OR ("variant"[All Fields] OR "variant s"[All Fields] OR "variants"[All Fields]) OR ("variation"[All Fields] OR "variations"[All Fields]) OR ("mutate"[All Fields] OR "mutated"[All Fields] OR "mutates"[All Fields] OR "mutating"[All Fields] OR "mutation"[MeSH Terms] OR "mutation"[All Fields] OR "mutations"[All Fields] OR "mutation s"[All Fields] OR "mutational"[All Fields] OR "mutator"[All Fields] OR "mutators"[All Fields]) OR ("socioaffect neurosci psychol"[Journal] OR "snp"[All Fields])))	153
Cochrane Library	#1 (IL-6):ti,ab,kw OR (Interleukin-6):ti,ab,kw<br>#2 (polymorphism):ti,ab,kw OR (variant):ti,ab,kw OR (variation):ti,ab,kw OR (mutation):ti,ab,kw OR (SNP):ti,ab,kw<br>#3 (asthma):ti,ab,kw OR (asthmatic):ti,ab,kw OR (atopic dermatitis):ti,ab,kw OR (allergic rhinitis):ti,ab,kw<br>#4 #1 AND #2 AND #3	16
Web of science	#1 TOPIC: (IL-6) OR TOPIC: (Interleukin-6)<br>Databases= WOS, CSCD, KJD, MEDLINE, RSCI, SCIELO Timespan=All years<br>Search language=Auto<br>#2 TOPIC: (polymorphism) OR TOPIC: (variant) OR TOPIC: (variation) OR TOPIC: (mutation) OR TOPIC: (SNP)<br>Databases= WOS, CSCD, KJD, MEDLINE, RSCI, SCIELO Timespan=All years<br>Search language=Auto<br>#3 TOPIC: (asthma) OR TOPIC: (asthmatic) OR TOPIC: (atopic dermatitis) OR TOPIC: (allergic rhinitis)<br>Databases= WOS, CSCD, KJD, MEDLINE, RSCI, SCIELO Timespan=All years<br>Search language=Auto<br>#4 #3 AND #2 AND #1<br>Databases= WOS, CSCD, KJD, MEDLINE, RSCI, SCIELO Timespan=All years<br>Search language=Auto	325

Supplementary Table 2. Results of pooled ORs in the meta-analysis of the association between *IL-6* gene polymorphisms and overall allergic disease. （After removing the studies of deviation from HWE）.

	Population	Sample size, cases/controls	Dominant comparison			Recessive comparison			Allele comparison			Heterozygote comparison			Homozygote comparison		
			p value	OR (95% CI)	I^2 statistic	p value	OR (95% CI)	I^2 statistic	p value	OR (95% CI)	I^2 statistic	p value	OR (95% CI)	I^2 statistic	p value	OR (95% CI)	I^2 statistic
	Overall	895/1262	0.33	0.74 [0.41, 1.35]	88%	0.32	0.73 [0.39, 1.35]	74%	0.35	0.81 [0.52, 1.26]	89%	0.38	0.79 [0.47, 1.34]	84%	0.24	0.58 [0.24, 1.44]	85%
**−174 G/C (rs1800795)**	Caucasian	432/765	0.10	0.62 [0.35, 1.10]	77%	0.60	0.81 [0.36, 1.79]	76%	0.09	0.69 [0.46, 1.06]	79%	0.11	0.67 [0.40, 1.10]	66%	0.13	0.49 [0.20, 1.22]	76%
	Asians	373/353	**0.03**	**1.53 [1.05, 2.23]**	**19%**	0.09	1.47 [0.94, 2.31]	NA	**0.02**	**1.39 [1.06, 1.83]**	**12%**	**0.03**	**1.43 [1.03, 1.98]**	**41%**	**0.04**	**1.67 [1.02, 2.74]**	**NA**
	Children	264/250	**P<0.0001**	**0.41 [0.28, 0.61]**	**NA**	**0.0005**	**0.47 [0.31, 0.72]**	**NA**	**P<0.00001**	**0.54 [0.42, 0.69]**	**NA**	**0.0006**	**0.48 [0.31, 0.73]**	**NA**	**P<0.00001**	**0.29 [0.18, 0.49]**	**NA**
	Adults	272/533	0.45	0.63 [0.19, 2.09]	90%	0.08	0.38 [0.13, 1.10]	0	0.47	0.73 [0.31, 1.72]	88%	0.53	0.71 [0.25, 2.03]	**89%**	**0.02**	**0.29 [0.10, 0.83]**	**0**

**−572 G/C (rs1800796)**	Overall	529/515	0.91	1.02 [0.78, 1.32]	0	0.09	1.43 [0.95, 2.17]	0	0.34	1.10 [0.90, 1.35]	0	0.59	0.92 [0.70, 1.23]	0	0.1	1.46 [0.93, 2.27]	0
**-597 G/A (rs1800797)**	Overall	311/719	0.36	0.88 [0.66, 1.16]	14%	0.80	1.16 [0.36, 3.74]	58%	0.72	0.94 [0.65, 1.35]	55%	0.25	0.84 [0.63, 1.13]	0	0.91	1.08 [0.31, 3.71]	62%
	Caucasian	311/719	0.36	0.88 [0.66, 1.16]	14%	0.80	1.16 [0.36, 3.74]	58%	0.72	0.94 [0.65, 1.35]	55%	0.25	0.84 [0.63, 1.13]	0	0.91	1.08 [0.31, 3.71]	62%
	Adults	74/301	0.08	0.63 [0.37, 1.06]	NA	0.22	0.47 [0.14, 1.59]	NA	0.06	0.66 [0.43, 1.01]	NA	0.16	0.68 [0.39, 1.16]	NA	0.15	0.40 [0.12, 1.38]	NA

Supplementary Table 3. Results of pooled ORs in the meta-analysis of the association between IL-6 gene polymorphisms and each allergic disease. （After removing the studies of deviation from HWE）

Polymorphism and disease	Population	Sample size, cases/controls	Dominant comparison			Recessive comparison			Allele comparison			Heterozygote comparison			Homozygote comparison		
			p value	OR (95% CI)	I^2 statistic	p value	OR (95% CI)	I^2 statistic	p value	OR (95% CI)	I^2 statistic	p value	OR (95% CI)	I^2 statistic	p value	OR (95% CI)	I^2 statistic
−174 G/C (rs1800795) and asthma	Overall	536/783	0.16	0.57 [0.26, 1.25]	**87%**	**0.0001**	**0.46 [0.31, 0.68]**	0	0.12	0.66 [0.39, 1.11]	83%	0.21	0.64 [0.32, 1.28]	**85%**	**P<0.00001**	**0.29 [0.18, 0.46]**	**0**
	Caucasian	338/551	**P<0.00001**	**0.46 [0.33, 0.63]**	**0**	**0.0003**	**0.47 [0.31, 0.70]**	**0**	**P<0.00001**	**0.52 [0.42, 0.63]**	**26%**	**0.0001**	**0.52 [0.37, 0.72]**	**0**	**P<0.000001**	**0.31 [0.19, 0.49]**	**0**
	Children	264/250	**P<0.00001**	**0.41 [0.28, 0.61]**	NA	**0.0005**	**0.47 [0.31, 0.72]**	**NA**	**P<0.00001**	**0.54 [0.42, 0.69]**	**NA**	**0.0006**	0.48 [0.31, 0.73]	**89%**	**P<0.000001**	**0.29 [0.18, 0.49]**	**NA**
	Adults	164/445	**0.02**	**0.67 [0.47, 0.94]**	90%	0.08	0.38 [0.13, 1.10]	0	0.47	0.73 [0.31, 1.72]	88%	**0.02**	**0.67 [0.48, 0.93]**	**89%**	**0.02**	**0.29 [0.10, 0.83]**	**0**

−174 G/C (rs1800795) and allergic rhinitis	Allergic rhinitis	265/265	0.09	1.36 [0.96, 1.94]	NA	0.09	1.47 [0.94, 2.31]	**NA**	**0.03**	**1.31 [1.02, 1.67]**	NA	0.24	1.26 [0.86, 1.84]	**NA**	**0.04**	**1.67 [1.02, 2.74]**	**NA**
−174 G/C (rs1800795) and atopic dermatitis	Atopic dermatitis	94/214	0.71	1.11 [0.65, 1.89]	NA	0.94	0.98 [0.54, 1.79]	NA	0.84	1.04 [0.73, 1.46]	NA	0.67	1.13 [0.64, 1.98]	NA	0.88	1.06 [0.52, 2.13]	NA
-597 G/A (rs1800797) and asthma	Overall	134/441	0.12	0.72 [0.48, 1.09]	0	0.14	0.43 [0.14, 1.32]	0	0.06	0.72 [0.50, 1.01]	0	0.26	0.79 [0.52, 1.20]	0	0.09	0.37 [0.12, 1.17]	0
	Caucasian	134/441	0.12	0.72 [0.48, 1.09]	0	0.14	0.43 [0.14, 1.32]	0	0.06	0.72 [0.50, 1.01]	0	0.26	0.79 [0.52, 1.20]	0	0.09	0.37 [0.12, 1.17]	0

Abbreviations: OR, Odds ratio; CI, Confidence interval.

The values in bold represent there is statistically significant differences between cases and controls.

Supplementary Table 4. *P* value of Egger's tests for *IL6* rs1800795 polymorphism and overall allergic diseases risk under the A: Dominant comparison; B: Recessive comparison; C: Allele comparison; D: Heterozygote comparison; E: Homozygote comparison.

Comparison	Dominant comparison	Recessive comparison	Allele comparison	Heterozygote comparison	Homozygote comparison
P value	0.775	0.723	0.773	0.907	0.886

Supplementary Figure 1. Pooled OR and 95% CI of individual studies and pooled data for the association between *IL-6* rs1800795 polymorphism and overall allergic diseases risk under the A: Dominant comparison; B: Recessive comparison; C: Allele comparison; D: Heterozygote comparison; E: Homozygote comparison.

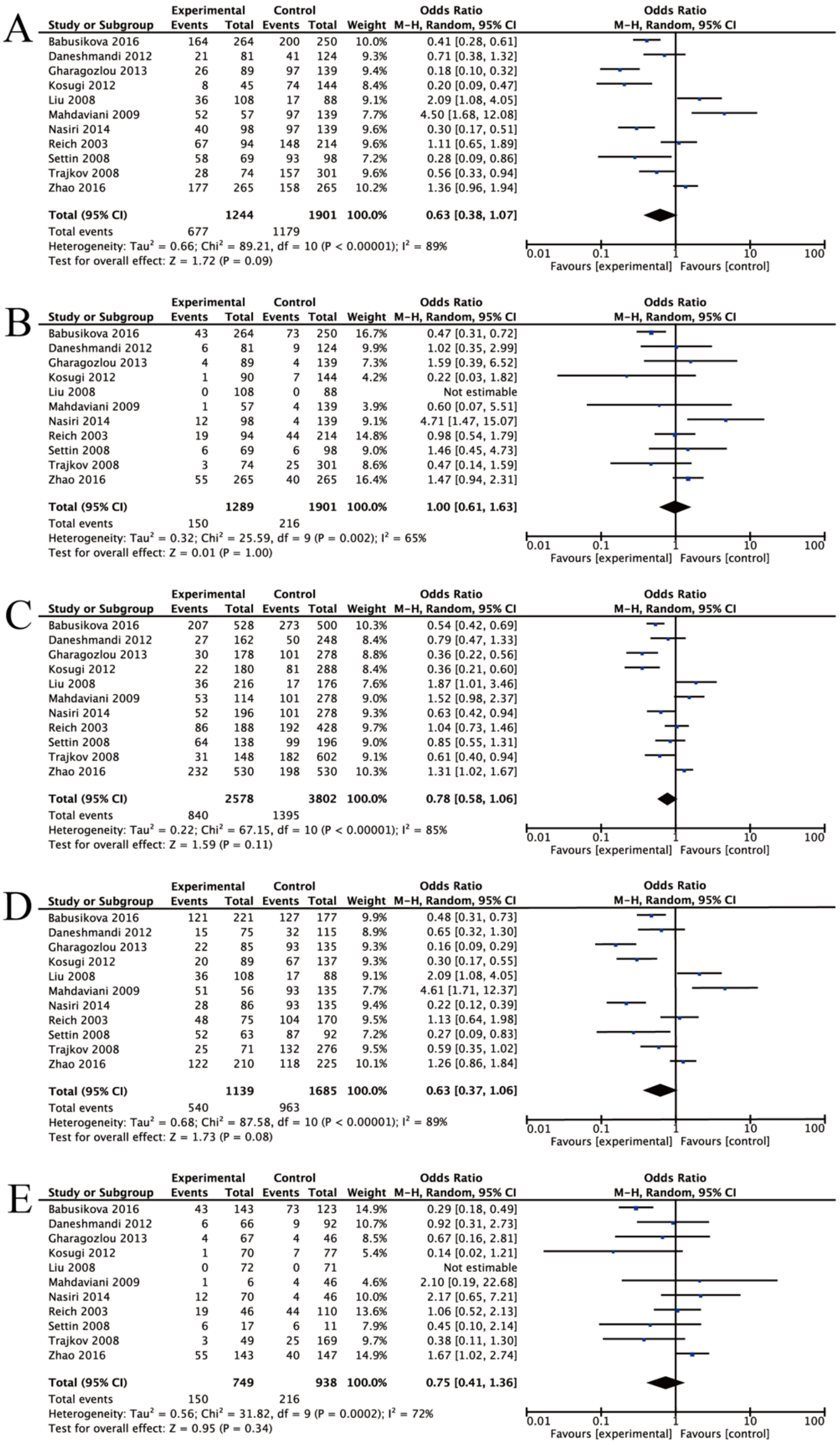

Supplementary Figure 2. Pooled OR and 95% CI of individual studies and pooled data for the association between *IL-6* rs1800796 polymorphism and overall allergic diseases risk under the A: Dominant comparison; B: Recessive comparison; C: Allele comparison; D: Heterozygote comparison; E: Homozygote comparison.

A

Study or Subgroup	Experimental Events	Experimental Total	Control Events	Control Total	Weight	Odds Ratio M-H, Fixed, 95% CI
Babusikova 2016	59	264	62	250	45.6%	0.87 [0.58, 1.31]
Zhao 2016	166	265	158	265	54.4%	1.14 [0.80, 1.61]
**Total (95% CI)**		**529**		**515**	**100.0%**	**1.02 [0.78, 1.32]**
Total events	225		220			

Heterogeneity: $Chi^2 = 0.92$, df = 1 (P = 0.34); $I^2 = 0\%$
Test for overall effect: Z = 0.12 (P = 0.91)

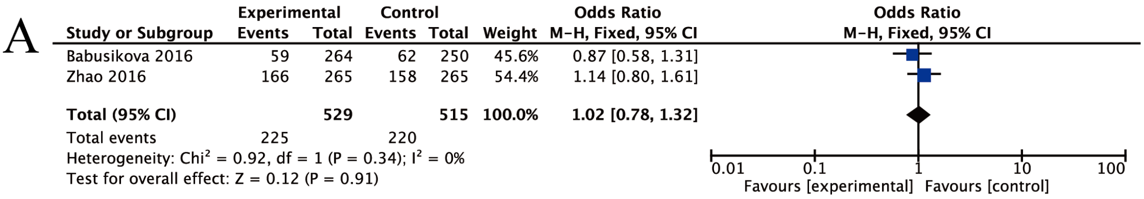

B

Study or Subgroup	Experimental Events	Experimental Total	Control Events	Control Total	Weight	Odds Ratio M-H, Fixed, 95% CI
Babusikova 2016	11	264	6	250	15.5%	1.77 [0.64, 4.86]
Zhao 2016	52	265	40	265	84.5%	1.37 [0.87, 2.16]
**Total (95% CI)**		**529**		**515**	**100.0%**	**1.43 [0.95, 2.17]**
Total events	63		46			

Heterogeneity: $Chi^2 = 0.20$, df = 1 (P = 0.65); $I^2 = 0\%$
Test for overall effect: Z = 1.71 (P = 0.09)

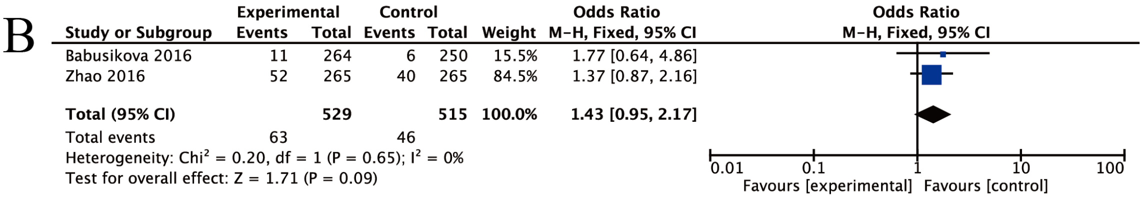

C

Study or Subgroup	Experimental Events	Experimental Total	Control Events	Control Total	Weight	Odds Ratio M-H, Fixed, 95% CI
Babusikova 2016	70	528	68	500	34.2%	0.97 [0.68, 1.39]
Zhao 2016	218	530	198	530	65.8%	1.17 [0.92, 1.50]
**Total (95% CI)**		**1058**		**1030**	**100.0%**	**1.10 [0.90, 1.35]**
Total events	288		266			

Heterogeneity: $Chi^2 = 0.71$, df = 1 (P = 0.40); $I^2 = 0\%$
Test for overall effect: Z = 0.95 (P = 0.34)

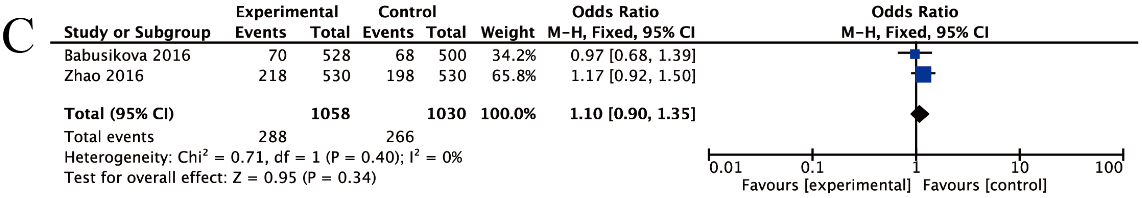

D

Study or Subgroup	Experimental Events	Experimental Total	Control Events	Control Total	Weight	Odds Ratio M-H, Fixed, 95% CI
Babusikova 2016	48	253	56	244	46.4%	0.79 [0.51, 1.21]
Zhao 2016	114	213	118	225	53.6%	1.04 [0.72, 1.52]
**Total (95% CI)**		**466**		**469**	**100.0%**	**0.92 [0.70, 1.23]**
Total events	162		174			

Heterogeneity: $Chi^2 = 0.94$, df = 1 (P = 0.33); $I^2 = 0\%$
Test for overall effect: Z = 0.54 (P = 0.59)

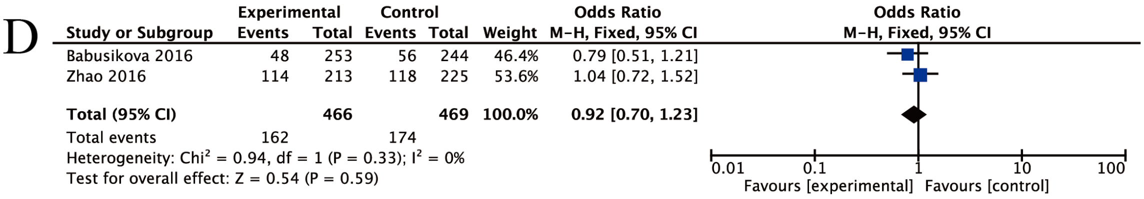

E

Study or Subgroup	Experimental Events	Experimental Total	Control Events	Control Total	Weight	Odds Ratio M-H, Fixed, 95% CI
Babusikova 2016	11	216	6	194	18.4%	1.68 [0.61, 4.64]
Zhao 2016	52	151	40	147	81.6%	1.41 [0.86, 2.30]
**Total (95% CI)**		**367**		**341**	**100.0%**	**1.46 [0.93, 2.27]**
Total events	63		46			

Heterogeneity: $Chi^2 = 0.10$, df = 1 (P = 0.76); $I^2 = 0\%$
Test for overall effect: Z = 1.66 (P = 0.10)

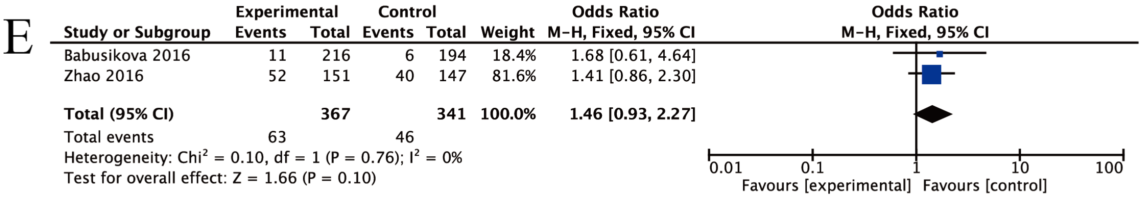

Supplementary Figure 3. Pooled OR and 95% CI of individual studies and pooled data for the association between *IL-6* rs1800797 polymorphism and overall allergic diseases risk under the A: Dominant comparison; B: Recessive comparison; C: Allele comparison; D: Heterozygote comparison; E: Homozygote comparison.

A

Study or Subgroup	Experimental Events	Experimental Total	Control Events	Control Total	Weight	Odds Ratio M–H, Fixed, 95% CI
Gharagozlou 2013	26	89	46	139	24.5%	0.83 [0.47, 1.49]
Liu 2008	0	108	0	88		Not estimable
Mahdaviani 2009	18	58	46	139	18.0%	0.91 [0.47, 1.76]
Nasiri 2014	38	98	46	139	22.5%	1.28 [0.75, 2.19]
Trajkov 2008	28	74	148	301	35.0%	0.63 [0.37, 1.06]
**Total (95% CI)**		**427**		**806**	**100.0%**	**0.88 [0.66, 1.16]**
Total events	110		286			

Heterogeneity: $Chi^2 = 3.50$, df = 3 (P = 0.32); $I^2 = 14\%$
Test for overall effect: Z = 0.92 (P = 0.36)

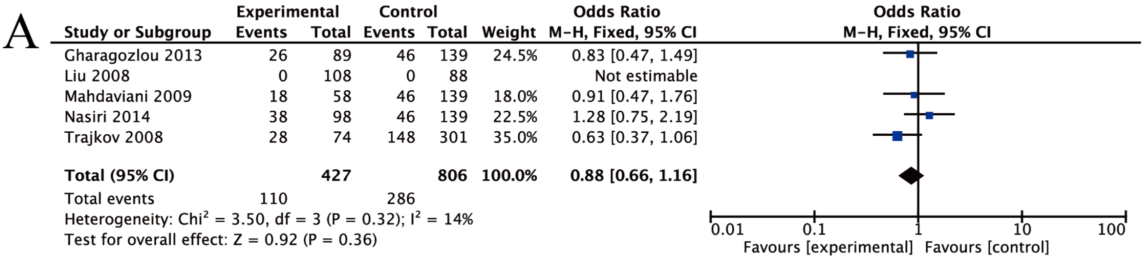

B

Study or Subgroup	Experimental Events	Experimental Total	Control Events	Control Total	Weight	Odds Ratio M–H, Random, 95% CI
Gharagozlou 2013	4	89	4	139	27.3%	1.59 [0.39, 6.52]
Liu 2008	0	108	0	88		Not estimable
Mahdaviani 2009	0	58	4	139	11.7%	0.26 [0.01, 4.86]
Nasiri 2014	10	98	4	139	30.8%	3.84 [1.17, 12.61]
Trajkov 2008	3	74	25	301	30.2%	0.47 [0.14, 1.59]
**Total (95% CI)**		**427**		**806**	**100.0%**	**1.16 [0.36, 3.74]**
Total events	17		37			

Heterogeneity: $Tau^2 = 0.79$; $Chi^2 = 7.18$, df = 3 (P = 0.07); $I^2 = 58\%$
Test for overall effect: Z = 0.25 (P = 0.80)

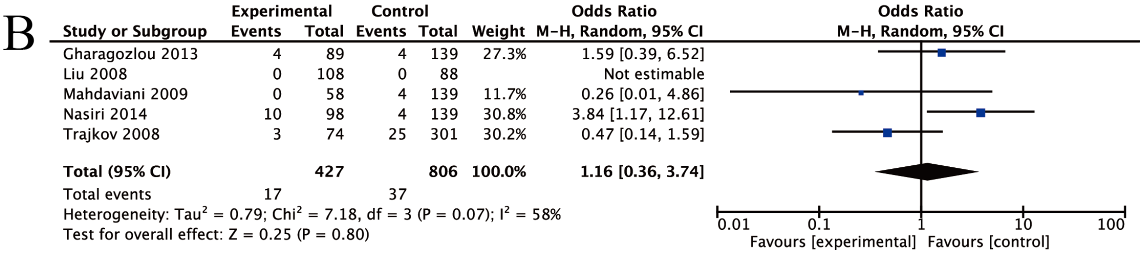

C

Study or Subgroup	Experimental Events	Experimental Total	Control Events	Control Total	Weight	Odds Ratio M–H, Random, 95% CI
Gharagozlou 2013	30	178	50	278	24.6%	0.92 [0.56, 1.52]
Liu 2008	0	216	0	176		Not estimable
Mahdaviani 2009	18	116	50	278	20.8%	0.84 [0.46, 1.51]
Nasiri 2014	48	196	50	278	27.0%	1.48 [0.95, 2.31]
Trajkov 2008	31	148	173	602	27.6%	0.66 [0.43, 1.01]
**Total (95% CI)**		**854**		**1612**	**100.0%**	**0.94 [0.65, 1.35]**
Total events	127		323			

Heterogeneity: $Tau^2 = 0.08$; $Chi^2 = 6.73$, df = 3 (P = 0.08); $I^2 = 55\%$
Test for overall effect: Z = 0.36 (P = 0.72)

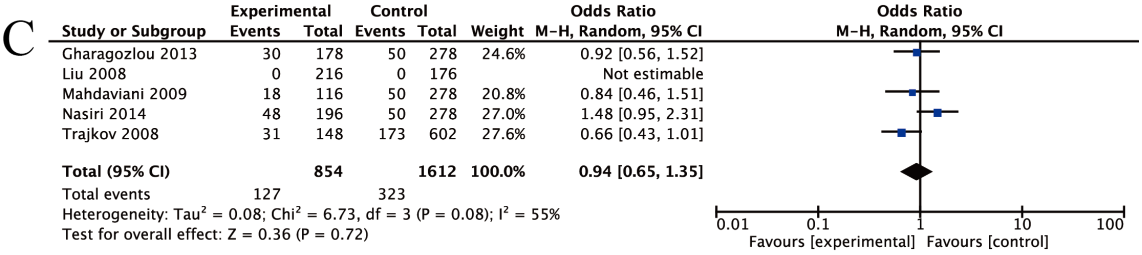

D

Study or Subgroup	Experimental Events	Experimental Total	Control Events	Control Total	Weight	Odds Ratio M–H, Fixed, 95% CI
Gharagozlou 2013	22	85	42	135	24.9%	0.77 [0.42, 1.42]
Liu 2008	0	108	0	88		Not estimable
Mahdaviani 2009	18	58	42	135	18.0%	1.00 [0.51, 1.94]
Nasiri 2014	28	88	42	135	23.4%	1.03 [0.58, 1.84]
Trajkov 2008	25	71	123	276	33.7%	0.68 [0.39, 1.16]
**Total (95% CI)**		**410**		**769**	**100.0%**	**0.84 [0.63, 1.13]**
Total events	93		249			

Heterogeneity: $Chi^2 = 1.44$, df = 3 (P = 0.70); $I^2 = 0\%$
Test for overall effect: Z = 1.14 (P = 0.25)

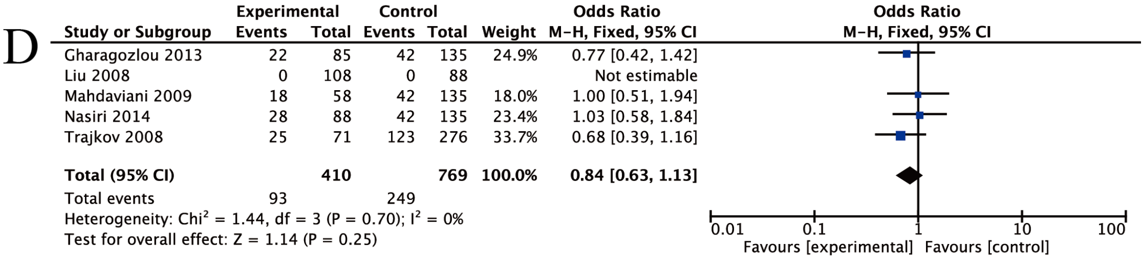

E

Study or Subgroup	Experimental Events	Experimental Total	Control Events	Control Total	Weight	Odds Ratio M–H, Random, 95% CI
Gharagozlou 2013	4	67	4	97	27.3%	1.48 [0.36, 6.12]
Liu 2008	0	108	0	88		Not estimable
Mahdaviani 2009	0	40	4	97	12.5%	0.26 [0.01, 4.88]
Nasiri 2014	10	70	4	97	30.4%	3.88 [1.16, 12.92]
Trajkov 2008	3	49	25	178	29.8%	0.40 [0.12, 1.38]
**Total (95% CI)**		**334**		**557**	**100.0%**	**1.08 [0.31, 3.71]**
Total events	17		37			

Heterogeneity: $Tau^2 = 0.94$; $Chi^2 = 7.88$, df = 3 (P = 0.05); $I^2 = 62\%$
Test for overall effect: Z = 0.12 (P = 0.91)

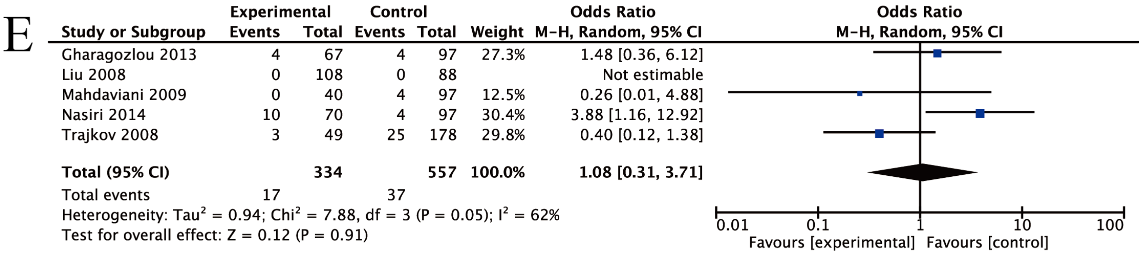

Supplementary Figure 4. Sensitivity analyses of *IL-6* rs1800795 and overall allergic diseases risk under the A: Dominant comparison; B: Recessive comparison; C: Allele comparison; D: Heterozygote comparison; E: Homozygote comparison.

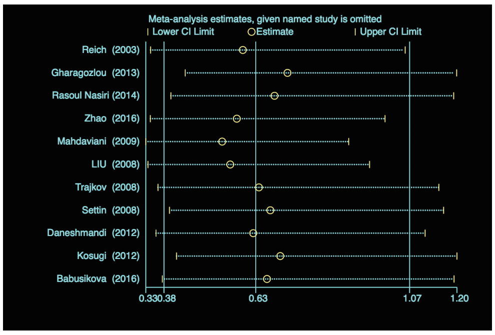

A

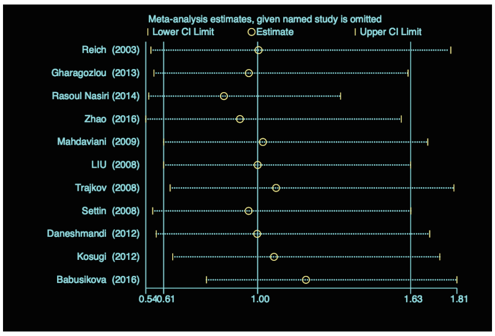

B

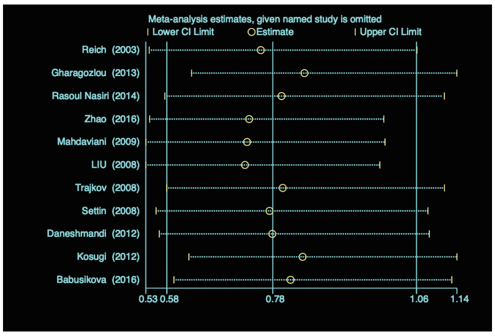

C

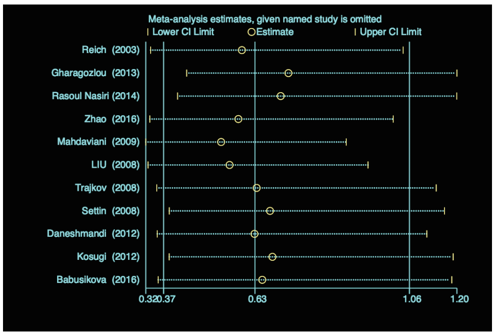

D

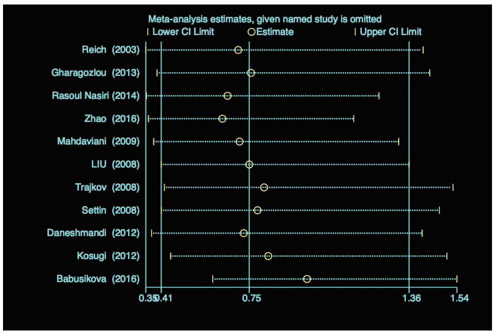

E

Supplementary Figure 5. Sensitivity analyses of *IL-6* rs1800797 and overall allergic diseases risk under the A: Dominant comparison; B: Recessive comparison; C: Allele comparison; D: Heterozygote comparison; E: Homozygote comparison.

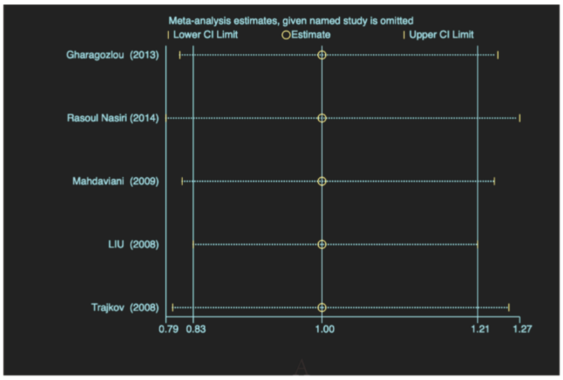

A

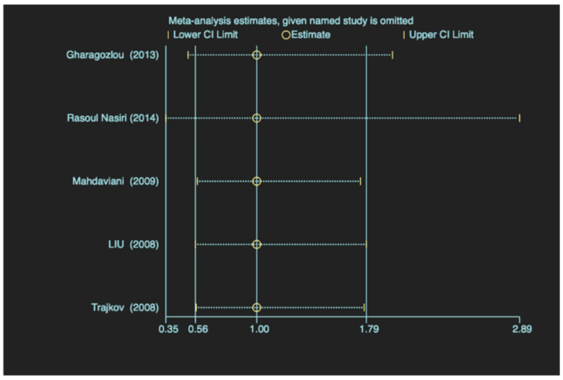

B

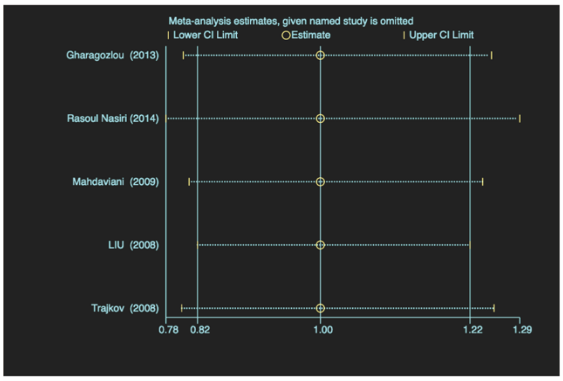

C

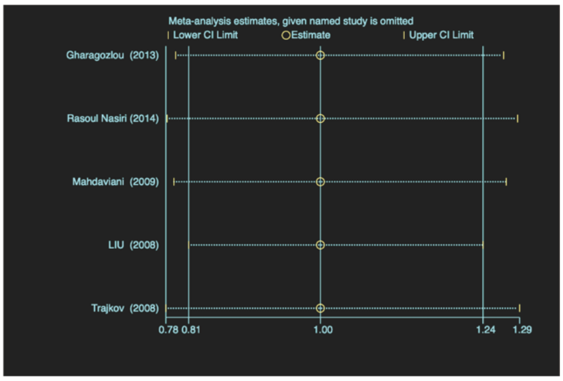

D

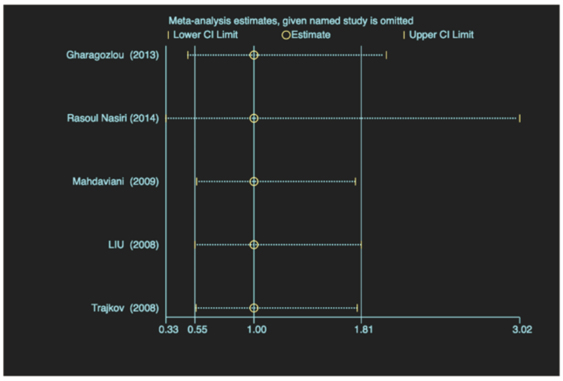

E

Supplementary Figure 6. Sensitivity analyses of *IL-6* rs1800795 and asthma risk under the A: Dominant comparison; B: Recessive comparison; C: Allele comparison; D: Heterozygote comparison; E: Homozygote comparison.

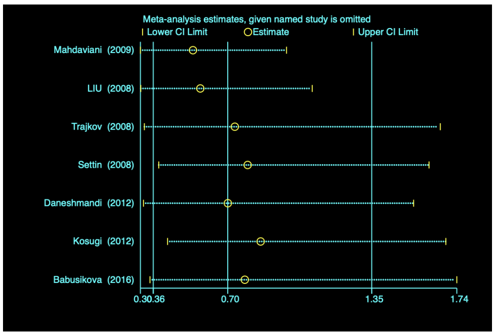

A

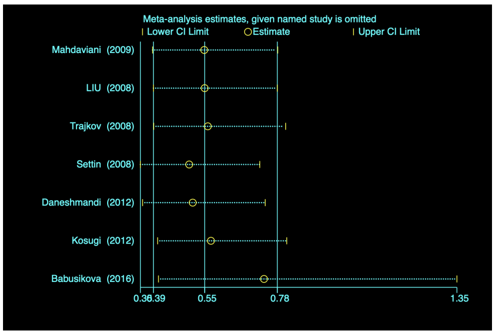

B

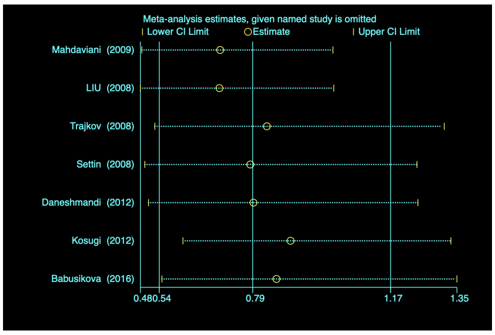

C

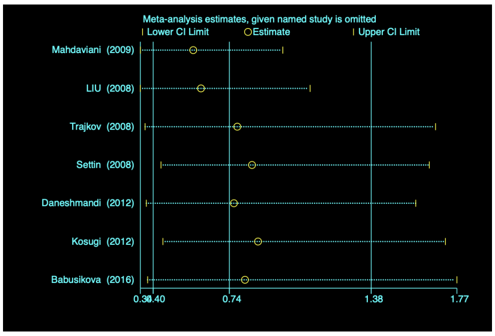

D

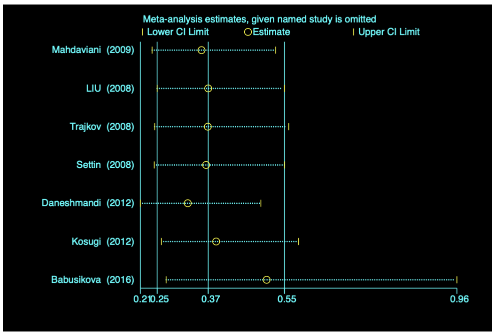

E

Supplementary Figure 7. Sensitivity analyses of *IL-6* rs1800797 and asthma risk under the A: Dominant comparison; B: Recessive comparison; C: Allele comparison; D: Heterozygote comparison; E: Homozygote comparison.

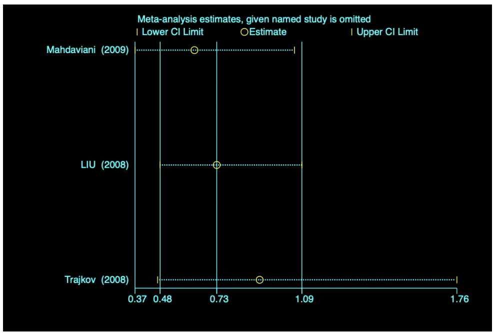

A

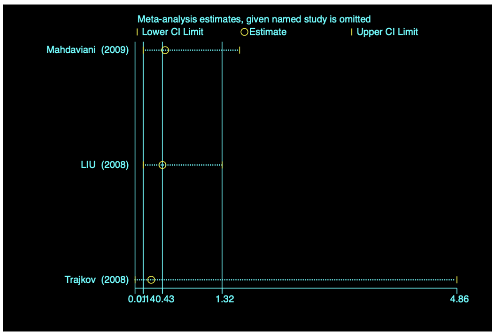

B

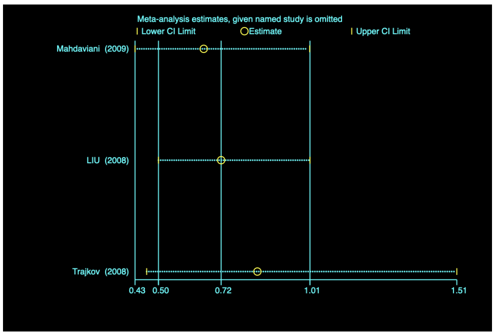

C

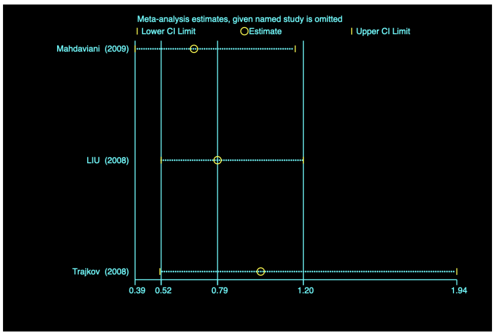

D

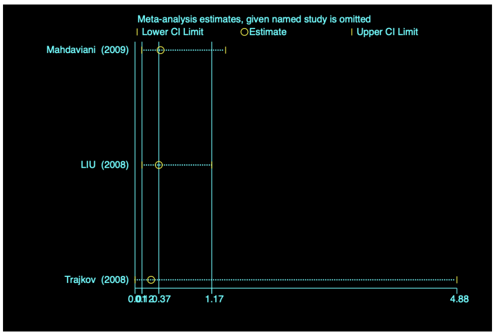

E

Supplementary Figure 8. Funnel plots of *IL6* rs1800795 and overall allergic diseases risk under the A: Dominant comparison; B: Recessive comparison; C: Allele comparison; D: Heterozygote comparison; E: Homozygote comparison.

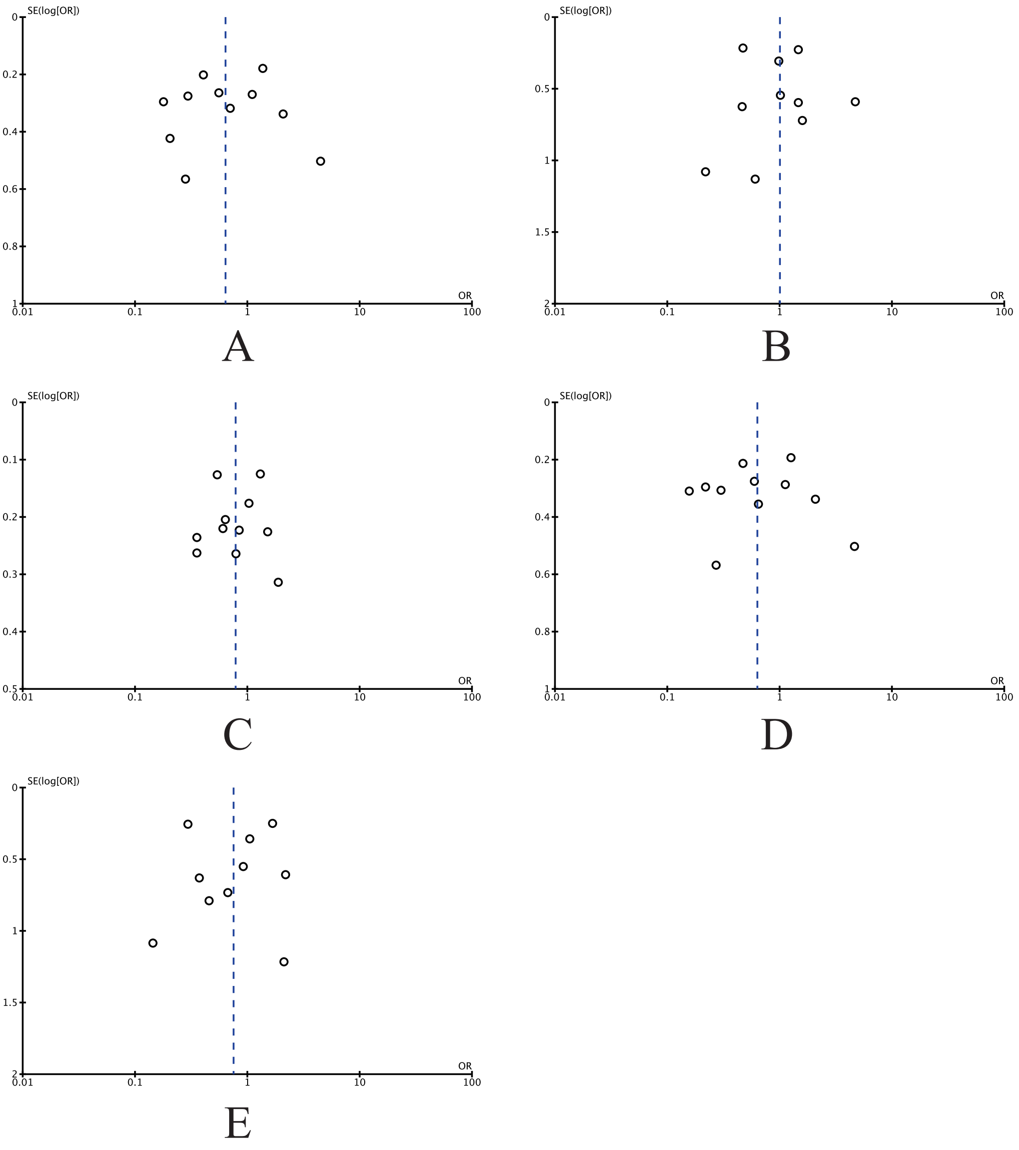

Supplementary Figure 9. Egger's tests for *IL-6* rs1800795 and overall allergic diseases risk under the A: Dominant comparison; B: Recessive comparison; C: Allele comparison; D: Heterozygote comparison; E: Homozygote comparison.

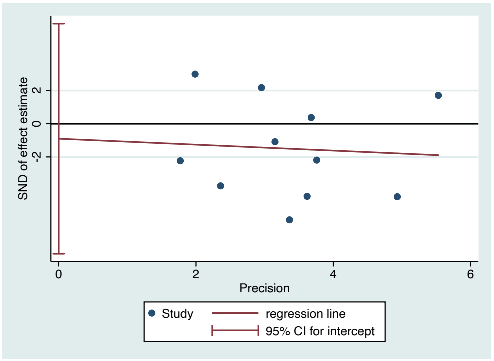

A

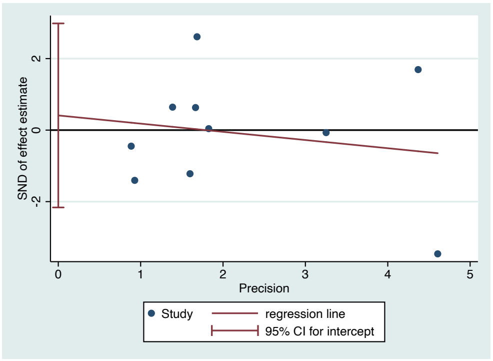

B

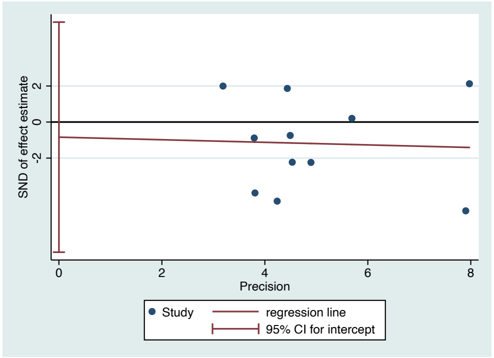

C

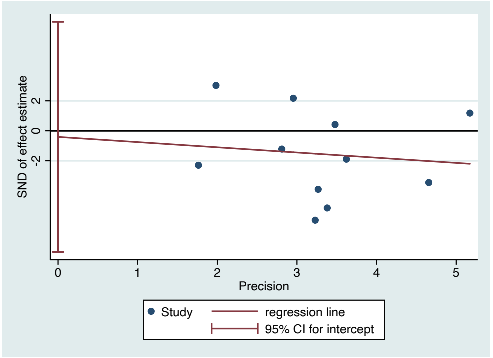

D

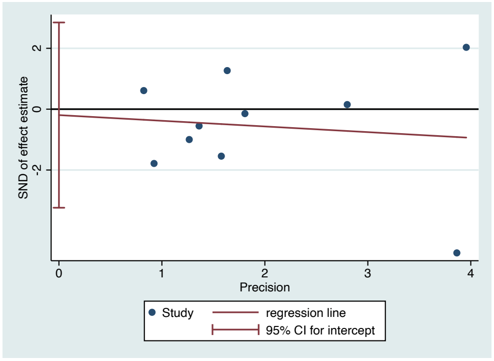

E

Supplementary Figure 10. The process of Egger's tests for *IL-6* rs1800795 and overall allergic diseases risk under the A: Dominant comparison; B: Recessive comparison; C: Allele comparison; D: Heterozygote comparison; E: Homozygote comparison.

. **metabias _ES _seES, egger graph**

Note: data input format ***theta se_theta*** assumed

Egger's test for small-study effects:
Regress standard normal deviate of intervention
effect estimate against its standard error

Number of studies = **11** Root MSE = **3.133**

Std_Eff	Coef.	Std. Err.	t	P>|t|	[95% Conf.	Interval]
slope	-.1791953	.8643343	-0.21	0.840	-2.134455	1.776065
bias	-.9016096	3.064687	-0.29	0.775	-7.834413	6.031194

Test of H0: no small-study effects P = **0.775**

A

. **metabias _ES _seES, egger graph**

Note: data input format ***theta se_theta*** assumed

Egger's test for small-study effects:
Regress standard normal deviate of intervention
effect estimate against its standard error

Number of studies = **10** Root MSE = **1.774**

Std_Eff	Coef.	Std. Err.	t	P>|t|	[95% Conf.	Interval]
slope	-.229227	.4345932	-0.53	0.612	-1.231401	.7729467
bias	.4102785	1.116147	0.37	0.723	-2.163562	2.984119

Test of H0: no small-study effects P = **0.723**

B

. **metabias _ES _seES, egger graph**

Note: data input format ***theta se_theta*** assumed

Egger's test for small-study effects:
Regress standard normal deviate of intervention
effect estimate against its standard error

Number of studies = **11** Root MSE = **2.718**

Std_Eff	Coef.	Std. Err.	t	P>|t|	[95% Conf.	Interval]
slope	-.0716735	.5404149	-0.13	0.897	-1.294177	1.15083
bias	-.8379512	2.821281	-0.30	0.773	-7.220133	5.54423

Test of H0: no small-study effects P = **0.773**

C

. **metabias _ES _seES, egger graph**

Note: data input format ***theta se_theta*** assumed

Egger's test for small-study effects:
Regress standard normal deviate of intervention
effect estimate against its standard error

Number of studies = **11** Root MSE = **3.116**

Std_Eff	Coef.	Std. Err.	t	P>|t|	[95% Conf.	Interval]
slope	-.3467979	.9881356	-0.35	0.734	-2.582116	1.88852
bias	-.4067153	3.394815	-0.12	0.907	-8.08632	7.27289

Test of H0: no small-study effects P = **0.907**

D

. **metabias _ES _seES, egger graph**

Note: data input format ***theta se_theta*** assumed

Egger's test for small-study effects:
Regress standard normal deviate of intervention
effect estimate against its standard error

Number of studies = **10** Root MSE = **1.991**

Std_Eff	Coef.	Std. Err.	t	P>|t|	[95% Conf.	Interval]
slope	-.1865779	.5799541	-0.32	0.756	-1.523954	1.150799
bias	-.1951114	1.320405	-0.15	0.886	-3.239971	2.849748

Test of H0: no small-study effects P = **0.886**

E
